# Optimizing communication strategies and designing a comprehensive program to facilitate cascade testing for familial hypercholesterolemia

**DOI:** 10.1186/s12913-023-09304-y

**Published:** 2023-04-05

**Authors:** Gemme Campbell-Salome, Laney K. Jones, Nicole L. Walters, Kelly M. Morgan, Andrew Brangan, Ilene G. Ladd, Mary P. McGowan, Katherine Wilemon, Tara J. Schmidlen, Emilie Simmons, Marci L. B. Schwartz, Megan N. McMinn, Eric Tricou, Alanna K. Rahm, Catherine D. Ahmed, Amy C. Sturm

**Affiliations:** 1 Department of Genomic Health, Geisinger, , Danville, PA USA; 2 Department of Population Health Sciences, Geisinger, Danville, PA USA; 3 Geisinger Heart and Vascular Institute, Geisinger, Danville, PA USA; 4 The Family Heart Foundation, Pasadena, CA USA; 5 Geisel School of Medicine at Dartmouth, Dartmouth Hitchcock Medical Center, Lebanon, NH USA; 6 Invitae, San Francisco, CA USA; 7 Cardiac Genome Clinic, Ted Rogers Centre for Heart Research, The Hospital for Sick Children, Toronto, ON Canada

**Keywords:** Familial hypercholesterolemia, Cascade testing, Chatbots, Direct contact, Implementation, Health communication

## Abstract

**Background:**

This project aimed to optimize communication strategies to support family communication about familial hypercholesterolemia (FH) and improve cascade testing uptake among at-risk relatives. Individuals and families with FH provided feedback on multiple strategies including: a family letter, digital tools, and direct contact.

**Methods:**

Feedback from participants was collected via dyadic interviews (*n* = 11) and surveys (*n* = 98) on communication strategies and their proposed implementation to improve cascade testing uptake. We conducted a thematic analysis to identify how to optimize each strategy. We categorized optimizations and their implementation within the project’s healthcare system using a Traffic Light approach.

**Results:**

Thematic analysis resulted in four distinct suggested optimizations for each communication strategy and seven suggested optimizations that were suitable across all strategies. Four suggestions for developing a comprehensive cascade testing program, which would offer all optimized communication strategies also emerged. All optimized suggestions coded green (*n* = 21) were incorporated. Suggestions coded yellow (*n* = 12) were partially incorporated. Only two suggestions were coded red and could not be incorporated.

**Conclusions:**

This project demonstrates how to collect and analyze stakeholder feedback for program design. We identified feasible suggested optimizations, resulting in communication strategies that are patient-informed and patient-centered. Optimized strategies were implemented in a comprehensive cascade testing program.

**Supplementary Information:**

The online version contains supplementary material available at 10.1186/s12913-023-09304-y.

## Background

Familial hypercholesterolemia (FH) is one of the most common genetic disorders, causing increased risk of premature atherosclerotic cardiovascular disease (ASCVD) [1, 2]; however, early diagnosis and treatment can significantly improve prognosis and be lifesaving [3]. Individuals with FH can be diagnosed through genetic testing of the main genes associated with FH (*LDLR*, *APOB*, *PCSK9*) and through clinical methods including low density lipoprotein (LDL) cholesterol testing, physical exam, and collection of a family health history [1, 4, 5]. Cascade testing, or the stepwise and systematic screening of at-risk relatives in the family, is an effective method of identifying additional individuals with FH, as most individuals with FH have an autosomal dominant form of the condition [6, 7]. However, FH cascade testing is not routinely performed in the U.S. and the burden of sharing risk information about FH and motivating family to pursue testing is left to the proband, or first person diagnosed with FH.

Probands report a myriad of challenges trying to communicate with their family about FH including difficulty recalling and sharing complex risk information, navigating geographic and emotional distance with at-risk relatives, and inability to motivate relatives to pursue diagnosis and treatment [8, 9]. Probands may be provided Dear Family Letters to share with at-risk relatives that aim to support family communication and cascade testing, but such passive methods remain suboptimal [10]. Recently, a systematic review found passive methods resulted in < 1 new relative with FH identified per proband on average [11]. Comparatively, more active methods such as clinicians directly contacting relatives resulted in a higher rate of new relatives with FH identified per proband [11]. Further, individuals with FH have expressed interest in receiving assistance from clinicians to share FH-related health risks with family [8, 9, 12].

Innovative, active communication strategies such as digital tools and direct contact are potential solutions to reduce the communication burden on probands and improve FH cascade testing uptake [13, 14]. Digital tools like chatbots can support patient activation by delivering standardized medical information designed by clinicians at the user’s pace and by increasing access to genetic counseling and testing resources [15, 16]. Chatbots are digital conversational agents that communicate in ways mirroring human dialogue and have been implemented in healthcare systems to scale the delivery of genetic information [15]. Direct contact is another novel, active method that has the potential to reduce proband burden and improve cascade testing uptake. Historically, programs outside the U.S. in which a clinician directly contacts at-risk relatives with a proband’s permission have been highly effective in identifying additional individuals in the family with FH [7, 11].

Chatbots and direct contact strategies can also help at-risk relatives navigate to cascade testing resources. Recent evidence found individuals with FH and clinicians described these novel communication strategies as both acceptable and appropriate as well as feasible to implement in current practice [17]. Moreover, there is evidence suggesting offering a combination of passive and active communication strategies to probands sharing an FH diagnosis with at-risk relatives can lead to a higher proportion of relatives being tested [18]. However, more research is needed to inform the development of a patient-centered program offering multiple communication strategies to FH probands to facilitate cascade testing [19].

This project aimed to gather perspectives from individuals and families with FH to optimize existing communication strategies (i.e., Dear Family Letter, chatbots) and design a new communication strategy (i.e., a direct contact program) to improve FH cascade testing uptake. We document feedback from participants on how to (re)design communication strategies and create a comprehensive cascade testing program offering the strategies to probands to facilitate their family communication and improve FH cascade testing uptake. We also describe how the transdisciplinary team with expertise in FH, pharmacy, genomic medicine, health communication, and implementation science incorporated participants’ feedback and what suggested optimizations the team could or could not feasibly incorporate. Results may inform other healthcare systems on how to develop, optimize, and incorporate innovative, patient-centered communication strategies to facilitate cascade testing uptake.

## Methods

### Setting

The Identification Methods, Patient Activation, and Cascade Testing for FH (IMPACT-FH) research study took place at Geisinger, a central Pennsylvania-based integrated healthcare delivery system. The Geisinger healthcare system consists of multiple hospitals and outpatient facilities, serving approximately 1.5 million patients annually. Additionally, Geisinger offers a health insurance plan, which is synchronized with clinical decisions made within the healthcare system to ensure high-quality care is accessible and affordable to plan members (approximately a third of Geisinger patients). The (re)designed communication strategies were targeted to individuals with FH identified through Geisinger’s MyCode® Community Health Initiative (MyCode) [19]. MyCode is a population-based genomics project that includes electronic health records (EHRs) data as well as genomic data generated from exome sequencing [20]. MyCode also includes a genomic screening initiative that returns actionable genetic results (including FH results) to patient-participants, called the MyCode Genomic Screening and Counseling Program (GSCP) [21]. The MyCode GSCP is equipped with 14 genetic counselors, 2 medical geneticists, 4 genetic counseling assistants, administrative leadership support, and study support staff, all at varying levels of funded time and effort. Additionally, MyCode GSCP offers genetic counselling and provides patient-facing resources such as chatbots and detailed summaries of genomic findings as well as provider-facing resources to help explain results and next steps. Finally, Geisinger has a multidisciplinary lipid clinic (MDLC), staffed with a lipidologist, genetic counselor, and pharmacist [22]. The MDLC cares for individuals with severe lipid disorders, such as FH, within the health system. Qualitative findings from this study were applied to create a comprehensive cascade testing program (i.e., IMPACT-FH Cascade Testing Program**)** to offer the optimized communication strategies to probands receiving an FH result from MyCode to facilitate family communication and cascade testing uptake.

### Design

This project used a parallel mixed method design (interviews and surveys) to gather feedback from individuals and families with FH to optimize communication strategies to improve FH cascade testing uptake. The combination of methods ensured the project team could identify different perspectives on FH and cascade testing, characterize family dynamics relevant to offering optimized strategies, and triangulate findings that capture the breadth and depth of stakeholder feedback [23, 24]. Employing multiple methods to collect feedback also enabled wider recruitment and participation than solely conducting interviews.

The current project is part of a larger mixed-method study, “Identification Methods, Patient Activation, and Cascade Testing for Familial Hypercholesterolemia (IMPACT-FH)”, that aims to examine the optimized communication strategies’ effectiveness in facilitating FH cascade testing uptake in a prospective, pragmatic trial [19]. This project follows the Standards for Reporting Qualitative Research (SRQR) [25]. The interview guide and survey were developed for this study by the transdisciplinary research team and have not been published elsewhere (see Supplementary Materials for interview guide and surveys).

### Data collection

We used a combination of purposive and snowball sampling to recruit participants. To be eligible, participants had to be (a) English speaking, and (b) diagnosed with FH through genetic testing or clinical criteria, (c) an at-risk family member, and/or (d) a family member (i.e. spouse) of someone with FH [26]. Eligible participants were invited to either complete a dyadic interview or respond to an online survey. Eligible participants were allowed to only participate in one method (either dyadic interview or survey).

Participants were recruited from Geisinger’s MyCode (MyCode) [20, 21], Geisinger’s MDLC [22], and via the Family Heart Foundation. The Family Heart Foundation is a national patient-centered research and advocacy organization that works to improve identification and care of genetic lipid disorders including FH and elevated Lipoprotein(a).

To complete dyadic interviews, the participant with an FH diagnosis was invited to and asked to recruit a family member to join. Participants who completed interviews received a $20 Amazon gift card. Participants were invited to complete surveys via email and through posts on the Family Heart Foundation’s social media accounts. Survey participants could also invite their family members to complete a survey. Survey participants recruited from Geisinger were entered into a raffle to win one of five $50 Amazon gift cards.

Participants of each method were asked to review existing communication strategies including a Dear Family Letter (Supplemental Fig. 1a), a Family Sharing Tool (FST) [27] for the proband to utilize, [27] and a Cascade Chatbot for relatives to receive and use. The FST is a flat webpage for probands to encourage communication of their FH result to family and allows them to send a Cascade Chatbot to their relatives (Supplemental Fig. 2a). The Cascade Chatbot is received by relatives and discusses the proband’s result, associated health risks for relatives, and recommended care for at-risk relatives (Supplemental Fig. 3a) [16]. Participants were provided a description on how clinicians could directly contact at-risk relatives with the proband’s permission. Questions among both methods focused on gathering participants’ perspectives on (1) how to optimize the letter, FST, and Cascade Chatbot, (2) how to design the direct contact strategy, (3) how to offer low-cost genetic testing options, (4) what strategy or combination of strategies they would use and why, and (5) additional suggestions for how to facilitate cascade testing for at-risk relatives.

Dyadic interviews were conducted by phone and audio-recorded. Transcripts were de-identified, checked for accuracy, and analyzed by the team. Responses to demographic questions and open-ended survey responses were exported from the survey, de-identified, and checked for accuracy by ensuring there was only one response per IP address, before inclusion in the full data set. Open-ended survey responses were moved to a spreadsheet to organize responses to each survey question on how to improve and design the strategies. Open-ended survey responses were then segmented by the type of strategy they gave feedback on. Interview transcripts and open-ended survey responses were iteratively read and analyzed concurrently. Descriptive statistics for participant demographics were analyzed using SPSS version 26.

### Data analysis

Two authors (G.C.S., N.L.W) kept operational memos and de-briefed after each interview to discuss emergent themes, refine probes, and discuss when saturation was being reached. The two authors noted that saturation was being reached after the ninth dyadic interview as no new data was surfacing from interviews about using the strategies with family and feedback on how to optimize the strategies became repetitive of previous interviews. At this time two more dyadic interviews were scheduled and conducted as planned to ensure saturation of concepts and feedback was reached for interviews. Before closing the survey, open-ended survey responses were reviewed by the two authors, who determined that saturation had been achieved and responses added to insights from dyadic interviews [26, 28].

Interviews and open-ended survey data were thematically analyzed using the constant comparative method to identify key points of participant feedback on how to optimize each communication strategy and develop a comprehensive program offering the strategies to support FH cascade testing uptake [29]. The two authors engaged in first-cycle coding by independently open-coding three interview transcripts. The two coders met to review one another’s coding, discuss discrepancies, iterate on the coding approach, and develop a codebook [30]. They proceeded to secondary-cycle coding by iteratively reviewing transcripts and survey responses and meeting to discuss codes to identify patterns, organize, and synthesize codes [28]. To ensure rigor, the coding team was expanded during axial coding to iteratively define and refine themes, descriptions, and examples and systematically group themes under hierarchical categories [29]. All coders had access to independently review transcripts and open-ended survey data in each phase of coding. The transdisciplinary coding team (G.C.S., N.L.W., L.K.J., C.D.A., K.M.M., A.C.S) included experts in genomic and precision medicine, pharmacy, implementation science, FH diagnosis and care, and an individual with FH. The diversity of the coding team ensured credibility of analysis that represents multi-faceted, crystallized qualitative findings [31].

The final data analysis step was to demonstrate how participant feedback was incorporated to optimize communication strategies for patient-participants receiving an FH result from MyCode. The transdisciplinary team utilized a Traffic Light approach to categorize how feasible participants’ suggested optimizations were within the context of the project and healthcare setting [32]. Green lights represent optimizations that fully addressed the participants’ feedback and could successfully be made (were feasible) within our setting. Yellow lights represent optimizations that partially incorporated the participant feedback. Red lights represent optimizations that could not be incorporated.

## Results

Overall, 120 participants were included in the project (see Table 1 for participant demographics). Eleven family dyads (*n* = 22) completed joint phone interviews between July–August 2020. Additionally, 98 separate participants responded to surveys conducted August–September 2020. Qualitative data between interviews and surveys provided consistent feedback on topics reported below. Of note, survey participants tended to express more hesitancy about whether genetic testing was necessary compared to clinical methods for identifying FH. Most survey participants reported being diagnosed with FH via clinical methods (cholesterol/lipid testing, physical exam, and family history), with about 32% having had FH genetic testing.

**Table 1:** Participant demographics

Overall Sample (*N* = 120)	
Sex	Female (75%)
	Male (25%)
Highest Educational Attainment	Some high school/high school/GED (14.2%)
	Some college/trade/technical degree (15.8%)
	Associate’s degree (6.7%)
	Bachelor’s degree (35%)
	Post-graduate degree (27.5%)
	Prefer not to answer (0.8%)
FH Diagnosis/Risk Status	Diagnosed (90.8%)
	At-risk (5%)
	Spouse/Caregiver (4.2%)
Dyadic Interview (*N* = 22)	
Age Ranges	25 – 34 (18.2%)
	35 – 44 (13.6%)
	45 – 54 (22.7%)
	55 – 64 (13.6%)
	> 65 (31.8%)
Dyadic Relationships	Sisters (*n* = 3)
	Spouse (*n* = 2)
	Mother-Daughter (*n* = 3)
	Mother-Son (*n* = 2)
	Father-Daughter (*n* = 1)
Survey Responses (*N* = 98)	
Participant Type	Individual with FH from Geisinger (*n* = 19, 19.4%)
	Individual with FH from the Family Heart Foundation (*n* = 72, 73.5%)
	Family member of an individual with FH (*n* = 7, 7.1%)
Age	14–80 years old (*M* = 55.94, *SD* = 13.45)

### Optimizing the Dear Family Letter resulted in a Family and Healthcare Professional Packet

Participants described finding the letter useful overall but recommended making printed and digital copies of the letter available, clarifying how to use the genetic testing report provided as part of the letter, incorporating edits to make the letter more personal so it might grab a relative’s attention, and eliminating jargon (Table 2). Using the Traffic Light approach, all suggested optimizations were categorized as green and incorporated except for the recommendation to remove logos. This suggestion was categorized as yellow because the letter still needed to identify the participating organizations. Based on these participant responses and recommendations to clarify the genetic testing report and make the letter more personal, we expanded the Dear Family Letter into a Family and Healthcare Professional Packet (Supplemental Figs. 1a and 1b).

**Table 2:**
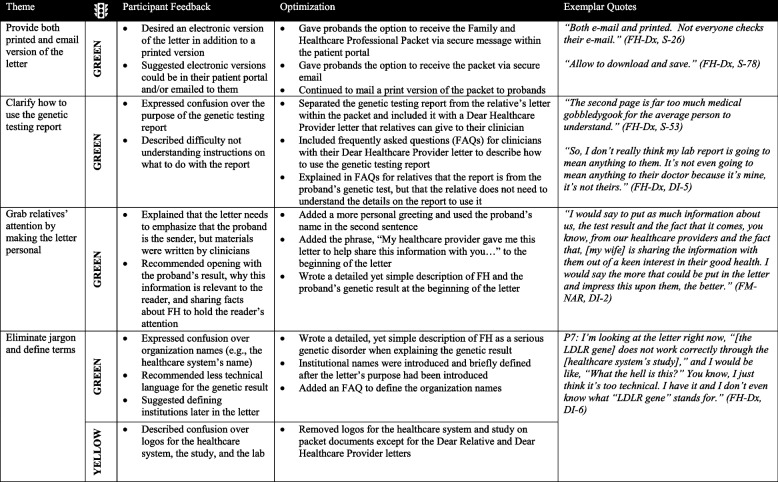
Optimizing the Dear Family Letter resulted in a Family and Healthcare Professional Packet

### Optimizing the Family Sharing Tool resulted in a Family Sharing Chatbot and Cascade Chatbot with new functionality

Participants described the FST and Cascade Chatbot as easy-to-use tools that could help at-risk relatives learn about FH in a non-threatening format. Participants recommended expanding the FST to be more interactive. They also recommended including a genetic testing ordering module in the Cascade Chatbot. These recommendations would enhance the technical capabilities of both tools. Participants also discussed their perceptions of the ideal types of users for chatbots (Table 3). Using the Traffic Light approach, four suggested optimizations were categorized as green (i.e., expand the FST, include an ordering module in the Cascade chatbot, offering additional options for sending a Cascade chatbot, and describing the chatbots as easy to use despite age/comfort with technology), two were yellow (i.e., Cascade chatbot reminders and overcoming perceptions that younger family members would prefer a chatbot), and one was red (i.e., offering a live chat function). The FST was expanded into a Family Sharing Chatbot (FSC) based on participants’ feedback on making the FST more interactive, similar to the Cascade Chatbot (Supplemental Figs. 2a and 2b). A genetic testing ordering module was added to the Cascade Chatbot based on participants’ recommendations to make it simple and accessible for at-risk relatives to pursue cascade testing for FH (Supplemental Fig. 3b).

**Table 3:**
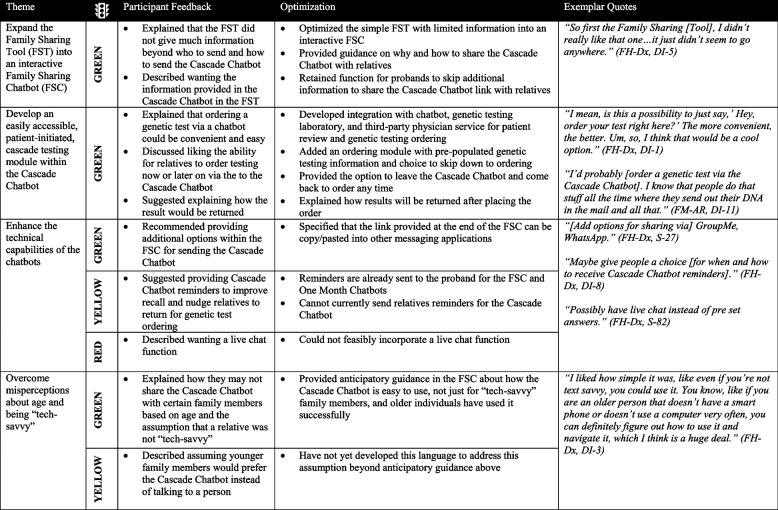
Optimizing the family sharing tool resulted in a family sharing chatbot and cascade chatbot with new functionality

### Designing the FH Outreach and Support Program for direct contact

Participants described how a clinician directly contacting their at-risk relatives to share information about FH and cascade testing could potentially motivate their family members to act. To create an acceptable direct contact strategy, participants recommended that an expert in FH who has a connection to the proband conduct the direct contact, relatives should be primed via a letter before direct contact takes place, clinicians within the same healthcare system of the proband and at-risk relative(s) should automatically coordinate contact without consent from the proband, and probands should be given the opportunity to partner with clinicians for direct contact (Table 4). Using the Traffic Light approach, two participant recommendations were categorized as green (i.e., having an expert with a connection to the proband and good communication skills perform direct contract, priming relatives before direct contact), while the other four recommended optimizations were categorized as yellow (i.e., having a PCP perform direct contact, giving a specific timeline for direct contact, automatically contacting an at-risk relative’s clinician within the same healthcare system as the proband, active proband involvement in direct contact). The direct contact strategy was designed based on participant recommendations and formally named the FH Outreach and Support Program. As part of the program, genetic counselors who worked with the original FH proband were the clinicians performing direct contact. First, probands received a flyer describing the program to help them consider whether to choose direct contact to help inform their at-risk relatives about their FH result (Supplemental Fig. 4). If probands chose direct contact, a primer letter was sent to alert the proband’s relative that they would be contacted by a clinician (Supplemental Fig. 5).

**Table 4:**
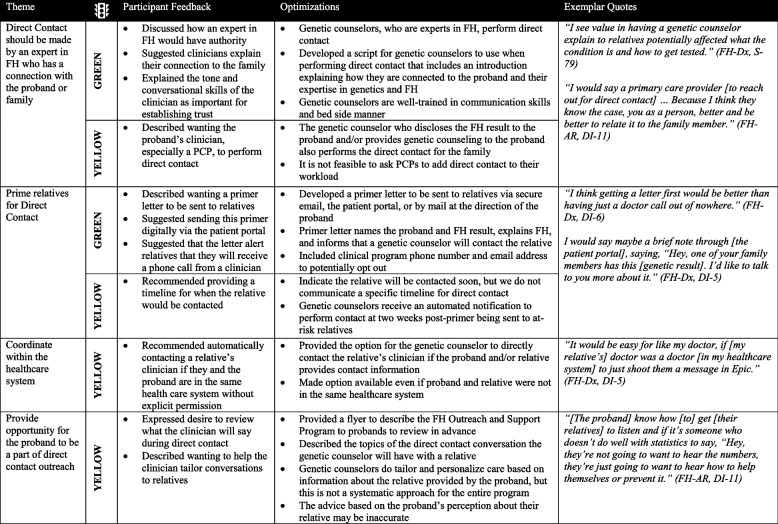
Designing the FH outreach and support program for direct contact

### Cross-cutting optimizations among strategies

Participants provided suggested optimizations that applied across strategies. Participants recommended providing credible, informative resources on FH for further information-seeking, including resources among strategies to help at-risk relatives navigate next action steps. This included clarifying the costs for cascade genetic testing and lipid testing, providing at-risk relatives with an option to connect directly with a clinician about their FH risks, stressing that the strategies are from a credible source and are trustworthy, improving strategies to get through the noise of spam/scams relatives may receive, and encouraging probands to give relatives a “heads up” before the strategy reaches the relative. Using the Traffic Light approach, seven participant recommendations were categorized as green, four recommendations were yellow, and one recommendation was red (Table 5). We implemented the green and yellow suggested optimizations across strategies, such as providing links to the Family Heart Foundation’s website for more information and resources, providing detailed instructions and multiple resources to help at-risk relative navigate next action steps, and using clear, transparent language comparing costs across options for cascade testing.

**Table 5:**
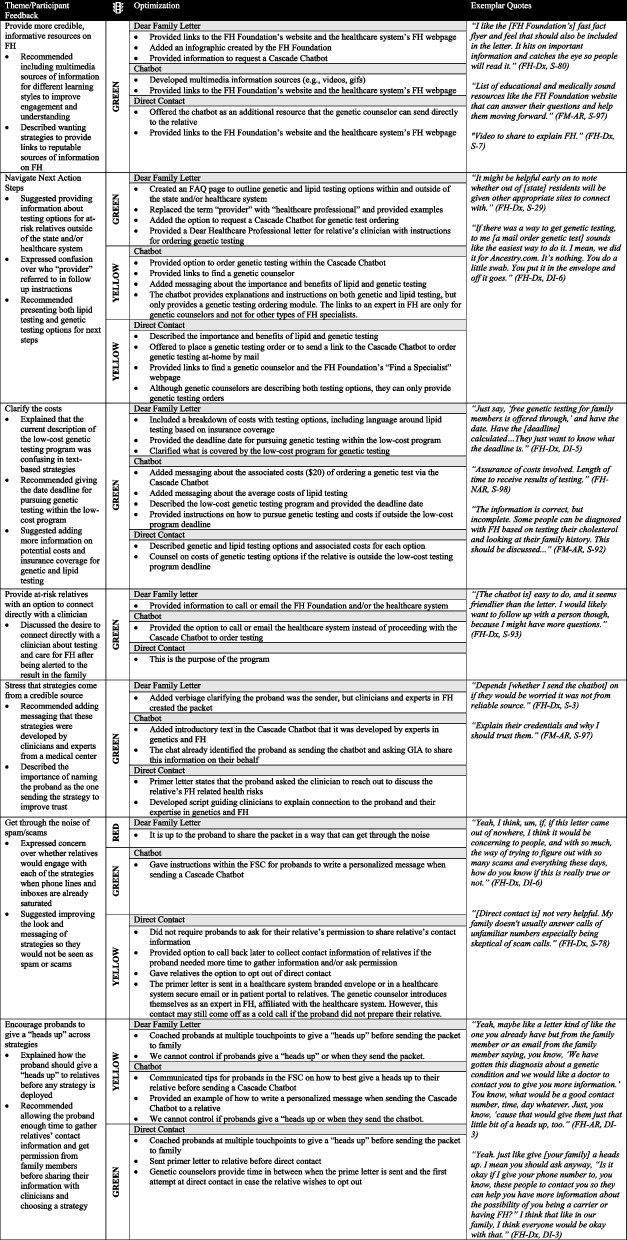
Cross-cutting optimizations among strategies

### Designing a comprehensive program—The IMPACT-FH Cascade Testing Program

As participants described their feedback on each communication strategy, they also described that they would like to use one or more of the strategies for each of their relatives as part of a comprehensive program. Participants recommended designing a multi-pronged program that provides options for probands regarding which strategies they could select to communicate their FH result with different family members. They also recommended offering the Cascade Chatbot to relatives within other strategies (i.e., QR code within packet), providing something tangible relatives could review and store (i.e., printed materials such as the packet, a transcript from the chatbot, direct contact primer letter), and encouraging future probands to tailor their strategy choices for each at-risk relative (Table 6). Using the Traffic Light approach, all suggested optimizations were categorized as green except for the suggestion that probands should contact family members to check their preferences to choose their preferred strategy, which was yellow. Based on this feedback, the team designed the IMPACT-FH Cascade Testing Program to provide the optimized, patient-centered strategies and provide probands choices among strategies over time. For the IMPACT-FH Cascade Testing Program, the team designed a workflow organizing how optimized strategies could function independently or in concert to facilitate family communication and cascade testing uptake (Supplemental Fig. 6).

**Table 6:**
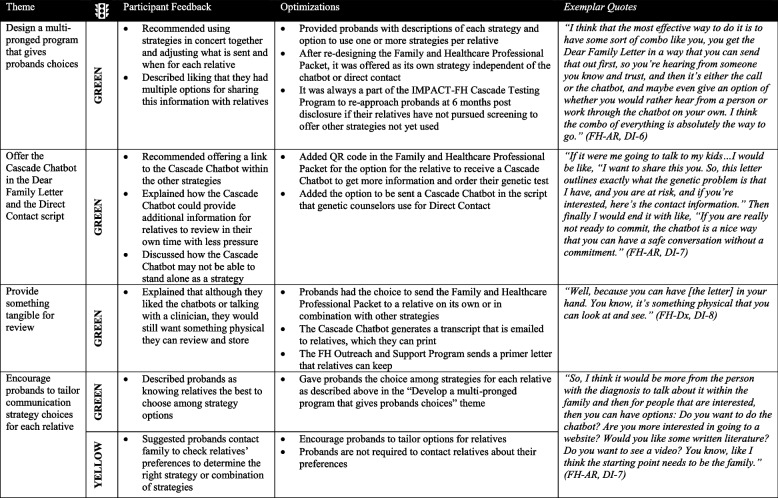
Designing a comprehensive program—the IMPACT-FH Cascade Testing Program

## Discussion

Developing a comprehensive program including patient-centered, innovative communication strategies can potentially overcome the challenges probands and their relatives face as they manage complex risk information about FH and pursue cascade testing [33]. Findings from this project identify key recommendations from individuals and families with FH to (re)design communication strategies and build a comprehensive cascade testing program. Using a Traffic Light approach [32], this project described how optimizations were mapped on to participant feedback and how optimizations could be feasibly incorporated within the study’s healthcare system. Finally, the process of analysis utilized in this project can inform similar approaches to collect critical feedback from the populations similar programs seek to serve. This approach also illustrates how to translate feedback to implement optimizations both in genomic and precision health as well as more broadly to areas of equity and inclusion efforts or community-based programs.

Incorporating feedback for optimizing the Dear Family Letter into a Family and Healthcare Professional Packet was relatively straightforward as most participant suggestions were to adjust the language and expand the single-page letter into a more comprehensive resource for relatives and their clinicians. Optimizations geared toward the relative’s clinician included a letter from a genetic counselor and an FAQ sheet notifying the clinician of the proband’s FH result, describing what the result could mean for the relative’s health, and providing simple steps for how the clinician could order cascade testing. Future research should examine how at-risk relatives and their clinicians respond to and use this type of resource. Recently, a web-based tool to enhance family communication by providing a digital letter for probands to share with relatives and educational modules was evaluated positively by individuals with FH and genetic counselors [14], which shows further promise for the optimizations made in this study. Additionally, while participants recommended expanding the letter into a packet, the additional information and length may seem overwhelming to some individuals and warrants further research. Overall, optimizations made to the letter alone may still face persistent limitations as it is a passive, proband-mediated strategy for family communication about FH and may need to be combined with other strategies to improve cascade testing uptake [10, 11].

Participants’ recommendations to expand the FST into a FSC and design a cascade test ordering module in the Cascade Chatbot were incorporated in partnership with the project’s healthcare system, a genetic testing laboratory, and a third-party physician ordering company [16, 19]. Incorporating a module within the Cascade Chatbot for a patient-initiated genetic testing order/mail-in genetic testing kit can improve access and ease of cascade testing uptake for at-risk relatives and improve FH diagnosis. With these optimizations chatbots can increase access to genetic testing and counseling resources [15]. However, some participant recommendations were not feasible to fully incorporate, such as reminders for relatives to return to the Cascade Chatbot if they start but do not complete the chat and including a live chat function with a human. For instance, developing a live chat would require an expert in FH be available any time a proband or relative opens the chatbot, which would significantly increase the workload of clinicians and their extenders (e.g., nurse, physician assistant). While our healthcare system and MyCode program cannot support a live chat due to limited availability of staff and experts in FH, other settings or environments equipped with larger staff trained by experts in FH may be able to feasibly facilitate a live chat with probands and relatives.

Although chatbots are more interactive strategies and can include direct access to genetic testing ordering, using the FSC to send a Cascade Chatbot to relatives still represents challenges inherent with proband-mediated communication strategies [11]. Probands may choose not to open or complete the FSC and not to send the Cascade Chatbot to relatives. Limitations to the reach and engagement with chatbots may also be due to technology access barriers. Although implementation of digital and mobile health tools are steadily increasing to fill gaps in healthcare, these tools can also increase disparities limiting their use to individuals who have technology skills and access to broadband connection to the Internet (i.e., the digital divide) [34]. It is promising that chatbots have been successfully integrated into the MyCode GSCP and participants in this study expressed interest in using and improving the chatbots [16, 35]. However, the digital divide and probands assumptions about relatives’ comfort with technology may limit the impact of the Cascade Chatbot to improve cascade testing uptake [11]. To address this limitation, our team added language to the FSC to combat these misperceptions. Relatives also can directly access the Cascade Chatbot as part of the IMPACT-FH Cascade Testing Program by requesting a Cascade Chatbot via contact information provided in the packet and/or when a genetic counselor performs direct contact.

Participant feedback on designing a direct contact program was implemented as much as feasibly possible to create the FH Outreach and Support Program. While participants often described wanting their primary care provider (PCP) to perform direct contact, they ultimately explained that the two most important characteristics were for the clinician to be an expert in FH and have a connection to the family. Asking PCPs to perform direct contact presents several key challenges including the lack of feasibility to incorporate direct contact into PCPs’ already full workload, lack of reimbursement for PCPs’ time, and limitations in their knowledge and confidence discussing genetic conditions and treating FH [17, 36, 37]. As such, genetic counselors were chosen to perform direct contact. Genetic counselors’ expertise is in line with qualities that participants desiring for the clinician performing direct contact, as genetic counselors are extensively trained in discussing genetic disorders, applying communication skills to disclose genetic risk information, and psychological support provision to patients and families. Other types of clinicians could perform direct contact if they are seen as experts and trusted sources of FH information and have strong communication skills, which could improve the feasibility of implementing direct contact programs widely [17]. Participants also recommended coordination of care within the healthcare system so the proband’s clinician could automatically share the FH diagnosis with at-risk relatives’ clinicians seamlessly, without first gaining permission from the proband. This suggested optimization represents ethical and legal challenges related to sharing private health information, as some clinicians may feel uncomfortable directly contacting a relative’s provider without the proband’s express permission, although it may be acceptable based on HIPAA requirements depending on state law [38, 39]. Beyond ethical and legal questions, system level barriers to clinician communication also limit wider implementation of this suggested optimization as not all clinicians and healthcare systems use EHRs that are interoperable to facilitate sharing risk information. Methods from implementation science can be utilized to design strategies for these complex care coordination programs that involve clinician to clinician communication regarding genetic information and to evaluate their effectiveness.

The Traffic Light approach is a method used in implementation science settings to explain how suggested adaptations have been incorporated into other projects [32, 40]. Utilizing the Traffic Light approach to describe suggested optimizations in this project improves the generalizability to other healthcare settings [32]. This approach facilitated the categorization of suggested optimizations based on their feasibility. Further, this approach demonstrates how to incorporate stakeholder feedback to optimize strategies and design healthcare programs. By assigning colors to suggested optimizations and describing the decisions for making and incorporating optimizations, other healthcare settings can decide what may be feasible for their patients, clinicians, and system.

A final key contribution of this work is the development of a comprehensive, multi-pronged program, the IMPACT-FH Cascade Testing Program, consisting of multiple optimized communication strategies to be utilized by probands receiving an FH result. Participants not only provided feedback on how to (re)design each communication strategy, but also provided recommendations on how to offer the strategies to probands, enable probands to choose a combination of strategies over time, and allow probands to customize their strategy choice(s) for each relative. Thus, the IMPACT-FH Cascade Testing Program was developed to describe and offer the optimized strategies to probands with FH, with the key tenet of allowing probands to tailor their choices for each at-risk relative and to switch strategies if the first choice did not work (Supplemental Fig. 6). Future research should examine how relatives respond to the proband’s strategy choices and use these optimized strategies to make decisions about their FH risk. Participant recommendations for offering a combination of passive and active communication strategies supports previous findings that offering multiple communication methods and types of clinical support can improve cascade testing uptake [18]. Future research should pragmatically test how such a program can improve cascade testing uptake and examine how probands and relatives use the optimized communication strategies [19] and what additional improvements can be made to these strategies.

Generalizability of this project is limited as the sample reported relatively high educational attainment, did not include non-English-speaking participants, and survey participants needed Internet access [41]. Further, all dyadic interview participants identified as Caucasian, and ethnicity and race were not collected in surveys. More diverse participants and those with lower educational attainment may experience meaningful differences when managing information about FH and pursuing cascade testing and could provide different suggested optimizations. As participants were recruited via Geisinger’s MyCode®, MDLC, and the Family Heart Foundation, they may have represented a group that is more active in pursuing information and testing for FH. Additionally, it is possible that some family members of interview participants completed survey responses, which may have created few instances with similar feedback due to a shared family communication experience or preferences. Although this is possible, authors took care during recruitment and data refinement to ensure survey responses were unique and that interview participants were not included in survey recruitment. Further, while our final optimized program and strategies are based on what was feasible within our healthcare system, others can review our data and utilize the Traffic Light approach to re-categorize suggested optimizations based on the resources available in their own system. For instance, Geisinger had genetic counselors return FH results to probands via MyCode® [21] and perform direct contact to at-risk relatives, and had certain facilitators (e.g., well-established genetic counseling program, previously established chatbot integration) that set the healthcare system up well to support optimizations. Finally, there may be other ways to establish a comprehensive cascade testing program outside of any one healthcare system, such as non-profit patient advocacy groups, that may address some of the implementation barriers encountered by this project.

## Conclusion

Overall, findings demonstrate the importance of offering choices to probands when providing patient-centered, innovative communication strategies to facilitate family communication about FH and cascade testing uptake. This project documents participant feedback to (re)design communication strategies and build a comprehensive patient-centered program to facilitate cascade testing uptake. Further, we demonstrate how feedback was implemented within the healthcare system and describe why some feedback could not be fully incorporated into the final optimized program. Other healthcare systems can learn from the Traffic Light approach to determine what feedback from participants can be feasibly implemented at their site to support family communication and FH cascade testing uptake. These learnings may inform family communication and cascade testing approaches for other genetic conditions.

## Supplementary Information

**Additional file 1: Supplemental Figure 1a.** Original Dear Family Letter. The original Dear Family Letter template with lab report for probands to share with at-risk relatives. **Supplemental Figure 1b.** Optimized Family and Healthcare Professional Packet. The optimized Dear Family Letter template with a flyer on FH, FAQs for relatives, a letter for the relative’s Healthcare Professional, and FAQs for the healthcare professionals.

**Additional file 2: Supplemental Figure 2a.** The original Family Sharing Tool (FST). The original FST with questions and answers for probands. The FST included a separate page to facilitate sharing of the Cascade Chatbot to at-risk relatives. **Supplemental Figure 2b.** The Family Sharing Chatbot (FSC). The FST was optimized into a FSC to be conversational and interactive chat to encourage probands to share information about their FH result with at-risk relatives.

**Additional file 3: Supplemental Figure 3a.** The Cascade Chatbot. The Cascade Chatbot is designed to share information about the proband’s FH result with at-risk relatives, provide the relative information about FH, and connect them with resources for cascade testing. **Supplemental Figure 3b.** The genetic testing ordering module additionto the Cascade Chatbot. The optimized Cascade Chatbot includes a module for at-risk relatives to order family variant testing through a mail-order genetic testing kit.

**Additional file 4: Supplemental Figure 4.** Flyer for the FH Outreach and Support Program. The flyer is sent to probands in a packet they receive after receiving their FH result from MyCode and describes important points related to the direct contact program.

**Additional file 5: Supplemental Figure 5.** Primer Letter for the FH Outreach and Support Program. The Primer Letter template for relatives the proband chooses to be contacted directly by a genetic counselor as part of the FH Outreach and Support Program.

**Additional file 6: Supplemental Figure 6.** IMPACT-FH Cascade Testing Program Workflow. Probands can choose multiple strategies for each of their at-risk relatives. All probands are provided with the Family and Healthcare Professional Packet and a flyer describing the FH Outreach and Support Program for direct contact after they receive their FH result even if they choose other communication strategies.

**Additional file 7.**

**Additional file 8.**

**Additional file 9.**

**Additional file 10.**

**Additional file 11.**

## Data Availability

The qualitative data that support the findings of this project are available on request from the corresponding author (G.C.S.). The data are not publicly available due to them containing information that could compromise participant privacy/consent.

## Abbreviations

FH: Familial Hypercholesterolemia
ASCVD: Atherosclerotic cardiovascular disease
LDL: Low density lipoprotein
IMPACT-FH: Identification Methods, Patient Activation, and Cascade Testing for Familial Hypercholesterolemia
SRQR: Standards for Reporting Qualitative Research
GSCP: Genomic Screening and Counseling Program
MDLC: Multi-disciplinary lipid clinic
FST: Family Sharing Tool
FSC: Family Sharing Chatbot
FAQ: Frequently Asked Questions
PCP: Primary care provider
