# Supplementary material for: Optimizing communication strategies and designing a comprehensive program to facilitate cascade testing for familial hypercholesterolemia
Source: BMC Health Serv Res. 2023 Apr 5;23:340. doi: 10.1186/s12913-023-09304-y (PMC10074725; doi:10.1186/s12913-023-09304-y)
Supplement: Supplementary file 1 — Additional file 1: Supplemental Figure 1a. Original Dear Family Letter. The original Dear Family Letter template with lab report for probands to share with at-risk relatives. Supplemental Figure 1b. Optimized Family and Healthcare Professional Packet. The optimized Dear Family Letter template with a flyer on FH, FAQs for relatives, a letter for the relative’s Healthcare Professional, and FAQs for the healthcare professionals. [file 12913_2023_9304_MOESM1_ESM.zip › Supplemental Figure 1a_Original LetterR4.pdf]

6/17/2020

Dear Ms. Jane Doe,

I learned that my *LDLR* gene does not work correctly through the Geisinger MyCode study. This gene change puts me at higher risk for early heart attack and stroke from inherited high cholesterol. Another name for this genetic risk is Familial Hypercholesterolemia (FH).

Gene changes run in families. Parents, children, brothers and sisters of people with a *LDLR* gene change have a 50% chance of having the same gene change and health risks. Other family members (aunts, uncles, nieces, nephews, cousins, grandchildren) may also have the same gene change and health risks.

People who have FH often need extra medical care. This extra care may include looking for and treating potentially life-threatening heart problems.

A simple “yes/no” blood or saliva test can tell you if you also have the same gene change and health risks.

You may be able to get this “yes/no” test for free or at lower-cost from the same lab that ran my test. Free genetic testing for family members is offered for 90 days after **6/10/2020**, my report date. The appointment with the provider to order this test will be billed to you or your insurance.

What you should do next:

- **Call the Geisinger MyCode Genomic Screening and Counseling team (toll-free) at 1-844-250-8031.** They can schedule an appointment to start the “yes/no” testing process. They can also answer your questions.

- **If you do not live in PA, you can find a genetic counselor to order this test and discuss your risks at:** https://www.nsgc.org/page/find-a-genetic-counselor

The healthcare provider ordering your test will need this information:

c.2054C>T p.Pro685Leu in the *LDLR* gene (NM_000527.5)
Laboratory: Invitae.

This letter gives my permission for Geisinger to share my genetic test results with you for your care. **Please bring this letter with you to your appointment.**

If you have any questions or concerns, please call the Geisinger MyCode team (toll-free) at **1-844-250-8031**.

Sincerely,

____
Mr. John Doe

mycode Geisinger

INVITAE

SECONDARY FINDINGS SCREEN RESULTS

**Patient name:**	John Doe	**Sample type:**	gDNA	**Report date:**	
**DOB:**		**Sample collection date:**		**Invitae #:**	
**Sex:**	Male	**Sample accession date:**		**Clinical team:**	
**MRN:**					

Test performed

Sequence analysis and deletion/duplication testing of the 59 genes listed in the Genes Analyzed section.

- Secondary Findings Add-on

RESULT: POSITIVE

A clinically significant genetic change was found in the LDLR gene, which is associated with a heart-related condition.

GENE	VARIANT	ZYGOSITY	VARIANT CLASSIFICATION
LDLR	c.2054C>T (p.Pro685Leu)	heterozygous	PATHOGENIC

About this test

This test evaluates 59 genes for variants (genetic changes) that indicate a significantly increased risk of developing certain types of cancer, heart-related conditions, or other types of actionable medical genetic conditions. These are disorders for which effective medical interventions and preventive measures are known and available. Genetic changes of uncertain significance are not included in this report; however, if additional evidence becomes available to indicate that a previously uncertain genetic change is clinically significant, Invitae will update this report and provide notification.

Next steps

- This is a medically important result that should be discussed with an appropriate healthcare provider. Genetic counseling is recommended to discuss the implications of this result and potential next steps.
- Consider sharing this result with relatives as they may also be at risk. Details on our Family Variant Testing program can be found at www.invitae.com/family.
- Register your test at www.invitae.com/patients to download a digital copy of your results. You can also access educational resources about how your results can help inform your health.

Clinical Summary

A Pathogenic variant, c.2054C>T (p.Pro685Leu), was identified in LDLR.
