# Supplementary material for: Optimizing communication strategies and designing a comprehensive program to facilitate cascade testing for familial hypercholesterolemia
Source: BMC Health Serv Res. 2023 Apr 5;23:340. doi: 10.1186/s12913-023-09304-y (PMC10074725; doi:10.1186/s12913-023-09304-y)
Supplement: Supplementary file 1 — Additional file 1: Supplemental Figure 1a. Original Dear Family Letter. The original Dear Family Letter template with lab report for probands to share with at-risk relatives. Supplemental Figure 1b. Optimized Family and Healthcare Professional Packet. The optimized Dear Family Letter template with a flyer on FH, FAQs for relatives, a letter for the relative’s Healthcare Professional, and FAQs for the healthcare professionals. [file 12913_2023_9304_MOESM1_ESM.zip › Supplemental Figure 1b_Optimized packetR4.pdf]

Geisinger

mycode

January 27, 2022

Dear ________________________,

I hope this letter finds you well. This is {Proband's Name}. I am sending you this letter because you are my relative and I want to share with you some important health information I recently learned that may also affect you. My healthcare professional gave me this letter to help me notify my relatives about this and to encourage you to take action.

**I was recently diagnosed with a serious genetic disorder called Familial Hypercholesterolemia (FH). FH is also known as inherited high cholesterol**. Having FH causes extremely high LDL ("bad") cholesterol levels from birth. Having high levels of bad cholesterol puts me at a **higher risk for early heart disease, heart attack, stroke, and possibly death, *<u>if left untreated</u>*.** Fortunately, there are effective treatment options available.

**FH runs in families.** This means parents, children, brothers, and sisters of people with FH have a **50% chance** of also having inherited FH or not. Other family members (aunts, uncles, nieces, nephews, cousins, grandchildren, etc.) may also have inherited this serious genetic disorder.

As my blood relative, **it is important that you be tested for FH for your own health.** I am not writing to scare you. I am writing because I care about you and I want everyone in our family to know if they have FH or not, and if they do, to get the best treatment available.

Most people with FH need specific medical care and treatment to significantly reduce their risk for heart disease, heart attack, stroke, and even early death. If you have FH, there are many effective treatment options available, including simple medications. **Starting treatment as early as possible can greatly reduce your risks.**

I found out that I have FH from a genetic test. I took the test through MyCode, a program at Geisinger. Geisinger is my healthcare system in Pennsylvania.

Learning if you have FH is the first step. A simple "yes/no" blood or saliva test can tell you if you also have inherited FH and the same health risks as me. **There is a program at Geisinger that can offer you a low-cost "yes/no" genetic test.** This test is available to my relatives who live anywhere. You do not have to be a Geisinger patient! People at risk for FH should also get a cholesterol test.

**The information in this packet can help you decide what to do next.**

**Included in this Packet:**

- An Information Sheet about FH
- Frequently Asked Questions (FAQs) and Suggested Next Steps to Take
- A letter to show to your healthcare professional, written by a Geisinger FH expert

I hope that you will follow up on this letter by taking action to learn if you too have FH or not. This letter gives my permission for Geisinger to share my FH-related healthcare information with you and your healthcare professional to support your care. If you have any questions or concerns, please contact the Geisinger team (toll-free) at **1 (844) 250-8031** or **MyCodeResults@geisinger.edu**.

Sincerely,

__________________________

{Proband's Name}

# Familial Hypercholesterolemia (FH)

## FH is Common

FH is a **genetic disorder** that causes dangerously **high levels of LDL ("bad") cholesterol** from birth, leading to early heart disease.

FH affects **1 in 250 people or 30 million worldwide** of all races and ethnicites.

FH is highly underdiagnosed - **90% of people with FH don't know they have it.**

## FH Causes Early Heart Disease

**~17,500** – the same number of people die from FH every year as from car accidents.

**~790,000 Americans** a year have a heart attack. Untreated individuals with FH have a **20X increased risk** of a heart attack.

## FH is Important to Find

Consider screening for FH if you have a family history of high cholesterol and/or early heart disease.

**FH can be diagnosed clinically or with a genetic test.**

Genetic testing for FH should include **pre- and post-genetic counseling**.

## FH Family

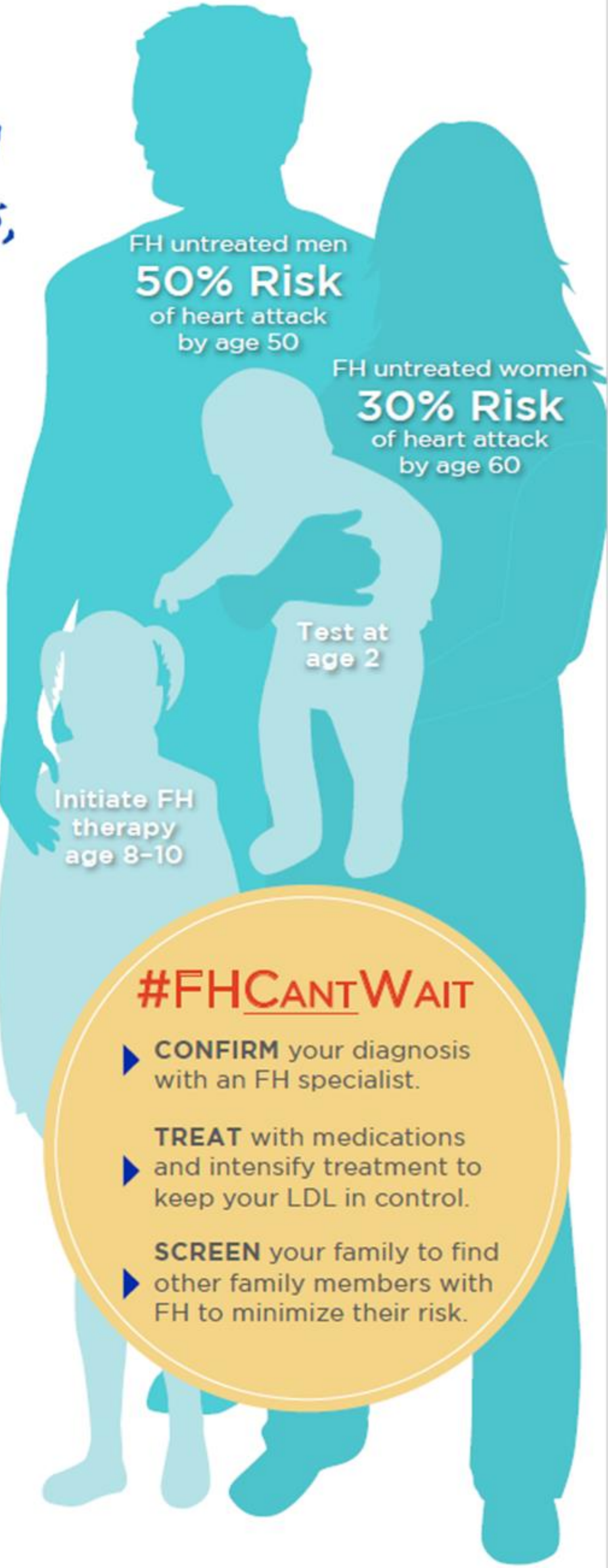


### #FHCantWait

- **CONFIRM** your diagnosis with an FH specialist.
- **TREAT** with medications and intensify treatment to keep your LDL in control.
- **SCREEN** your family to find other family members with FH to minimize their risk.

Learn more and get support at
**www.theFHfoundation.org**

FH FOUNDATION

a 501(c)(3) non-profit research and advocacy organization

© 2018, The FH Foundation. All rights reserved. 07/18

Raising Awareness. Saving Lives.

# FAQs

## Can You Tell Me More About Genetic Testing Through This Program?

**This simple "yes/no" genetic test is a clear way to determine if you also have inherited FH.**

{Proband's Name} received their FH result from Geisinger. Geisinger is a hospital system in Pennsylvania. Geisinger partners with Invitae to provide genetic testing to relatives of people who have tested positive for FH. Invitae is a national genetic testing lab.

- The genetic test provides families with an accurate diagnosis based on the first family member's result.
- You can get low-cost genetic testing through Invitae for a limited time.

The genetic test can be done on blood or saliva.

- An at-home mail-in kit can be used for a saliva sample.
- You can get a blood test from your healthcare professional.

The cost of your genetic test is covered **if ordered by {End Date of 150-Day Window} from Invitae**.
You and/or your insurance will pay costs if:

- You choose to order a genetic test from Invitae after this date.
- You choose to order a genetic test from a lab other than Invitae.
- You choose to visit a healthcare professional.

## Should I Also Have Cholesterol Testing?

**Yes, you should! A cholesterol test is a simple blood test that can help a healthcare professional find out if you have FH. Your healthcare professional can monitor your cholesterol levels and risk.**

Regardless of whether you get a genetic test, we strongly recommend that you get your cholesterol checked.

- Talk to your healthcare professional about ordering a cholesterol test that includes an LDL-C level.
- You and/or your insurance will pay for any costs associated with the cholesterol test or visits with a healthcare professional.

# FAQs

## What Can I Do Next?

**Here is what you can do to find out if you have FH.**

Please note that these are ***not*** your only options. These are ways that can help you get a low-cost genetic test from Invitae and/or a cholesterol test that includes an LDL-C level.

**You can even pick multiple options – just choose what works best for you!**

| Options | *If you live...* | |
|---|---|---|
| | **Inside Pennsylvania** | **Outside Pennsylvania** |
| **A** **You can see a Geisinger healthcare professional.**<br>• **Set up** an in-person or virtual visit with your healthcare professional of choice at Geisinger.<br>• You can meet with a:<br>  o Genetic counselor, who is an FH expert and worked with your family member.<br>    ▪ Call **1 (844) 250-8031** to schedule a visit.<br>  o Primary care provider.<br>  o Cardiologist, a doctor who specializes in the heart.<br>  o Lipidologist, a doctor who specializes in cholesterol and blood fats.<br>• **Discuss** getting a genetic test and a cholesterol test that includes an LDL-C level at your visit.<br>• **Bring** the attached letter to your visit. Show the letter to your healthcare professional.<br>  o This letter contains a copy of your family member's genetic testing lab report. Your healthcare professional will need this if you choose to order a low-cost genetic test with Invitae.<br>• **You and/or your insurance will pay for costs due to:**<br>  o Ordering genetic testing outside of the low-cost window.<br>  o Ordering from a lab other than Invitae during the low-cost window.<br>  o Cholesterol tests.<br>  o Visits with a healthcare professional. | ✓ | ✗ |
| **B** **You can see a non-Geisinger healthcare professional.**<br>• **Set up** an in-person or virtual visit with your healthcare professional of choice outside of Geisinger.<br>• You can follow up with your healthcare professional or use the links below to find an FH specialist near you.<br>  o Find an FH specialist in your area at **www.thefhfoundation.org/find-fh-specialist**.<br>  o Find a genetic counselor in your area at **www.nsgc.org/page/find-a-genetic-counselor**.<br>• **Discuss** getting a genetic test and a cholesterol test that includes an LDL-C level at your visit.<br>• **Bring** the attached letter to your visit. Show the letter to your healthcare professional.<br>  o This letter contains a copy of your family member's genetic testing lab report. Your healthcare professional will need this if you choose to order a low-cost genetic test with Invitae.<br>• **You and/or your insurance will pay for costs due to:**<br>  o Ordering genetic testing outside of the low-cost window.<br>  o Ordering from a lab other than Invitae during the low-cost window.<br>  o Cholesterol tests.<br>  o Visits with a healthcare professional. | ✓ | ✓ |
| **C** **You can order the "yes/no" genetic test yourself!**<br>• **Call or email our team** to request a link to order your low-cost genetic test online.<br>  o Phone Number: **1 (844) 250-8031**<br>  o Email Address: **MyCodeResults@geisinger.edu**<br>  o You can receive the ordering link via text message, email, or MyGeisinger.<br>  o In your message, include your name, date of birth, and how you would like to receive the link (mobile phone number, email address, or MyGeisinger).<br>• **Place your order** using the link. You will receive a mail-in testing kit that requires a saliva sample.<br>  o **There will be a small fee of $20 to order your genetic test with this option.**<br>• In addition to ordering the genetic test, you should follow up with a healthcare professional to discuss getting a cholesterol test that includes an LDL-C level.<br>• **If you are outside of the time window for the low-cost genetic testing with Invitae, please call a Geisinger genetic counselor (free of charge) at 1 (844) 250-8031 to discuss testing options.** | ✓ | ✓ |

# FAQs

## What If I Have Questions About FH Or This Program?

**It is important to understand this information and why this is so important for you and your family. We are here to help!**

The **Geisinger Team** is here to help provide more information about FH and answer your questions about this program.

***Email***
MyCodeResults@geisinger.edu

***Phone***
1 (844) 250-8031

***Geisinger FH Webpage***
geisinger.org/FH

The **FH Foundation** is a patient-centered organization that can help answer your questions about FH, provide support, and connect you with others with FH.

***Email***
info@theFHfoundation.org

***Phone***
1 (626) 583-4674

***Website***
theFHfoundation.org

## What Is The Attached Letter For My Healthcare Professional?

**The attached letter can help you discuss this information and these testing options with your healthcare professional.**

Share the letter with your healthcare professional to inform them of your FH risk and discuss getting a genetic test and/or a cholesterol test that includes an LDL-C level.

- This letter also includes a copy of {Proband's Name}'s genetic testing lab report. Your healthcare professional will need this letter and lab report if you choose to have them order your genetic test with Invitae.
- You may review these materials as well, but do not worry if you do not understand them. Your healthcare professional will use it and can call our team if they have any questions.

## Optional Research Study

**You have an opportunity to participate in a research study.**

This choice is optional and ***<u>not</u>*** required for you to pursue testing. This study would ask for you to share test results that you may get.

**If you are interested in hearing more about this optional research study, please contact the study team. When reaching out, just refer to the "IMPACT-FH study"!**

***Email***
MyCodeResults@geisinger.edu

***Phone***
1 (844) 250-8031

Geisinger mycode

January 27, 2022

Dear Healthcare Professional,

**Your patient's relative was recently diagnosed with Familial Hypercholesterolemia (FH).**

**FH is a genetic condition** that causes very high lifelong LDL-cholesterol levels and is associated with increased risk for heart disease, heart attack, stroke, and premature death, if left untreated. First-degree relatives have a **50% chance** of having FH. More distant relatives are also at risk. If left untreated, people with FH have **20 times** the risk of developing premature heart disease. **Risk of premature death and cardiovascular events, such as heart attack and stroke, can be reduced by as much as 80% with early, aggressive therapy.**[1]

Your patient's relative received a positive result for FH through genetic testing via MyCode, a community health initiative at Geisinger, a healthcare system in Pennsylvania. As FH is an inherited condition, **we ask that you consider ordering genetic testing for your patient.**

Through the MyCode program, the cost of your patient's genetic test is covered by Invitae if it is ordered by **{End Date of 150-Day Window}**, otherwise regular fees will apply.

The following pages will give you more information about Geisinger, Invitae, the MyCode program, and how to place an order for the genetic test. **Included with this information is your patient's relative's genetic testing lab report.**

We also strongly suggest that your patient has their **LDL-cholesterol checked, regardless of whether genetic testing is pursued.**

If you have any questions or concerns, please do not hesitate to contact us at **1 (844) 250-8031** or **MyCodeResults@geisinger.edu**.

Sincerely,

Amy C. Sturm

Amy Curry Sturm, MS, CGC
**Professor**
**Licensed, Certified Genetic Counselor**
Genomic Medicine Institute
**Co-Director,** MyCode Genomic Screening and Counseling Program
**Director,** Cardiovascular Genomic Counseling
(570) 214-2192 | asturm@geisinger.edu

[1] Versmissen J, Oosterveer DM, Yazdanpanah M, et al. Efficacy of statins in familial hypercholesterolaemia: a long term cohort study. *BMJ*. 2008;337:a2423. Published 2008 Nov 11. doi:10.1136/bmj.a2423

# Healthcare Professional FAQs

## Can You Tell Me More About Genetic Testing Through This Program?

**A genetic test is a clear way to determine if your patient has inherited FH.**

Your patient's relative received their FH result from Geisinger. Geisinger is a hospital system in Pennsylvania. Geisinger partners with Invitae, a nationwide genetic testing laboratory, to provide genetic testing.

- Your patient is eligible for low-cost genetic testing through Invitae for a limited time.

The genetic test can be done on blood or saliva.

- You can order a blood test or mail-in kit for your patient from Invitae's website.
- At-home mail-in kits can be used to collect the saliva sample.

The cost of your patient's Family Variant genetic test is covered by Invitae **if it is ordered by {End Date of 150-Day Window}**.

- If you order your patient's genetic test from Invitae after this date or at a different laboratory, regular fees will apply.
- Your patient and/or their insurance will be responsible for any potential costs associated with testing outside of this window or at a different laboratory.
- Your patient and/or their insurance will be responsible for any potential costs associated with visiting a healthcare professional.

## Does My Patient Also Need Cholesterol Testing?

**Yes, they do! A cholesterol panel that includes LDL-C can also help you determine if your patient has FH.**

Regardless of whether your patient gets a genetic test, we strongly suggest that they have their cholesterol checked.

- Consider talking to your patient about ordering a cholesterol test that includes an LDL-C level.
- Your patient and/or their insurance will be responsible for any potential costs associated with the cholesterol test or visits with a healthcare professional.

Cholesterol testing and visits with a healthcare professional will not be covered by this program.

- Your patient and/or their insurance will be responsible for any potential costs associated with the cholesterol test or visits with a healthcare professional.

# Healthcare Professional FAQs

## How Can I Order A Genetic Test For My Patient?

**The links below will give you step-by-step instructions on how to order your patient's Family Variant genetic test through Invitae.**

Please use Code "**IMPACTFH**" when ordering.

For any additional questions and ordering assistance, please contact **Invitae's client services**.

***Email***
ClientServices@invitae.com

***Phone***
1 (800) 436-3037

***Invitae's Online Ordering Instructions***
invitae.com/en/ordering

***Invitae's Family Follow-up Testing Information***
invitae.com/en/family

## What If I Have Questions About FH Or This Program?

**It is important to understand this information and why this is so important for your patient and their family. We are here to help!**

The **Geisinger Team** is here to help provide more information about FH and answer your questions about this program.

***Email***
MyCodeResults@geisinger.edu

***Phone***
1 (844) 250-8031

***Geisinger FH Webpage***
geisinger.org/FH

The **FH Foundation** is a patient-centered organization that can help answer your questions about FH and provide support for you and your patient.

***Email***
info@theFHfoundation.org

***Phone***
1 (626) 583-4674

***Website***
theFHfoundation.org

**The following page contains your patient's relative's Invitae lab report.**

**The information from the genetic test result will be needed to order your patient's genetic test.**

**Please use Code "IMPACTFH" when ordering.**

INSERT 1st PAGE OF PROBAND'S GENETIC TESTING REPORT HERE

| | | | | | |
|---|---|---|---|---|---|
| **Patient name:** | John Doe | **Sample type:** | gDNA | **Report date:** | |
| **DOB:** | | **Sample collection date:** | | **Invitae #:** | |
| **Sex:** | Male | **Sample accession date:** | | **Clinical team:** | |
| **MRN:** | | | | | |

**Test performed**

Sequence analysis and deletion/duplication testing of the 59 genes listed in the Genes Analyzed section.

- Secondary Findings Add-on

## RESULT: POSITIVE

**A clinically significant genetic change was found in the LDLR gene, which is associated with a heart-related condition.**

| GENE | VARIANT | ZYGOSITY | VARIANT CLASSIFICATION |
|---|---|---|---|
| LDLR | c.2054C>T (p.Pro685Leu) | heterozygous | PATHOGENIC |

**About this test**

This test evaluates 59 genes for variants (genetic changes) that indicate a significantly increased risk of developing certain types of cancer, heart-related conditions, or other types of actionable medical genetic conditions. These are disorders for which effective medical interventions and preventive measures are known and available. Genetic changes of uncertain significance are not included in this report; however, if additional evidence becomes available to indicate that a previously uncertain genetic change is clinically significant, Invitae will update this report and provide notification.

## Next steps

- This is a medically important result that should be discussed with an appropriate healthcare provider. Genetic counseling is recommended to discuss the implications of this result and potential next steps.
- Consider sharing this result with relatives as they may also be at risk. Details on our Family Variant Testing program can be found at www.invitae.com/family.
- Register your test at www.invitae.com/patients to download a digital copy of your results. You can also access educational resources about how your results can help inform your health.

## Clinical Summary

A Pathogenic variant, c.2054C>T (p.Pro685Leu), was identified in LDLR.
