# Supplementary figures and images for: Optimizing communication strategies and designing a comprehensive program to facilitate cascade testing for familial hypercholesterolemia
Source: BMC Health Serv Res. 2023 Apr 5;23:340. doi: 10.1186/s12913-023-09304-y (PMC10074725; doi:10.1186/s12913-023-09304-y)

**Figure 2a. The original Family Sharing Tool (FST).**


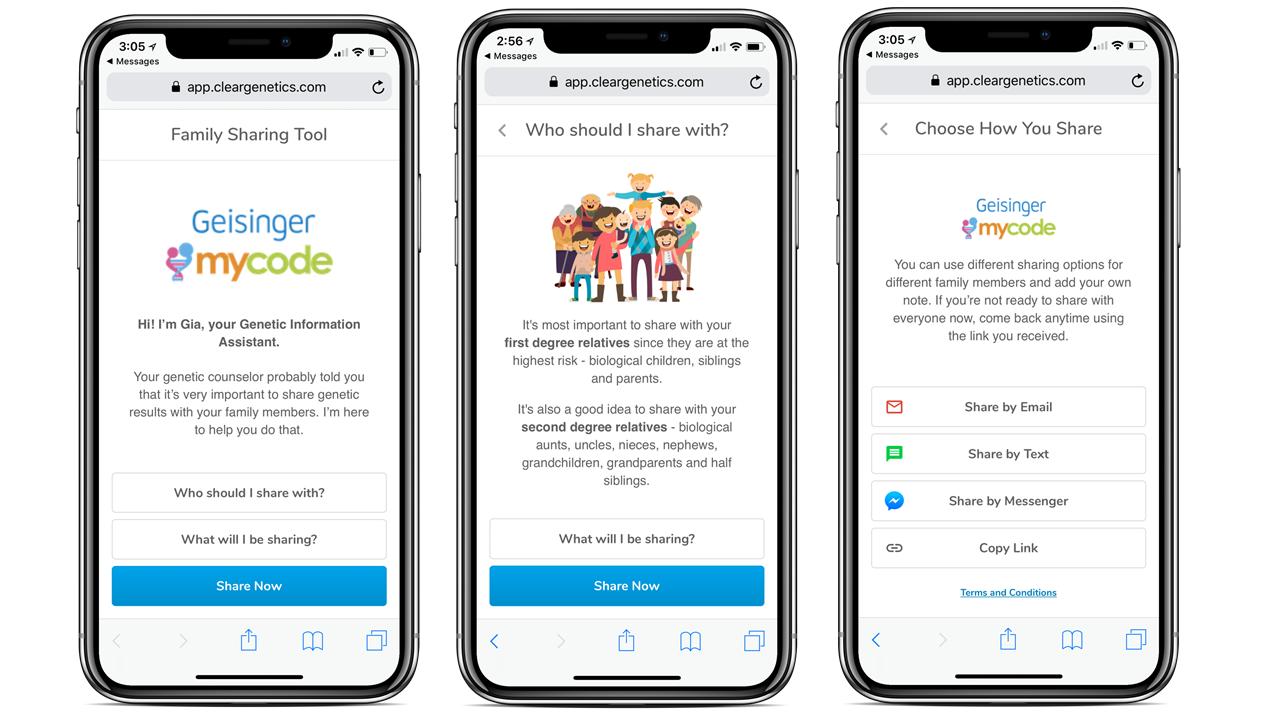

Supplement: Supplementary file 2 — Additional file 2: Supplemental Figure 2a. The original Family Sharing Tool (FST). The original FST with questions and answers for probands. The FST included a separate page to facilitate sharing of the Cascade Chatbot to at-risk relatives. Supplemental Figure 2b. The Family Sharing Chatbot (FSC). The FST was optimized into a FSC to be conversational and interactive chat to encourage probands to share information about their FH result with at-risk relatives. [file 12913_2023_9304_MOESM2_ESM.zip › Figure 2a_FSTR4.docx]

**Figure 2b. The Family Sharing Chatbot (FSC).**


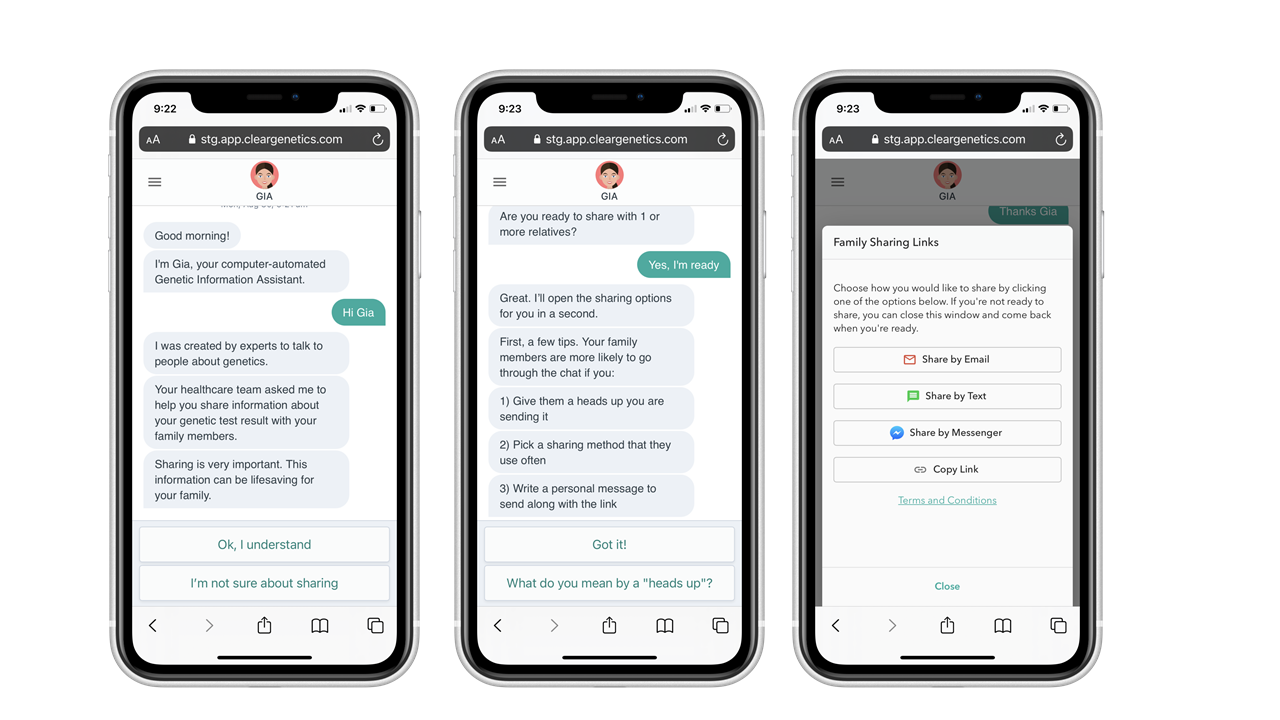

Supplement: Supplementary file 2 — Additional file 2: Supplemental Figure 2a. The original Family Sharing Tool (FST). The original FST with questions and answers for probands. The FST included a separate page to facilitate sharing of the Cascade Chatbot to at-risk relatives. Supplemental Figure 2b. The Family Sharing Chatbot (FSC). The FST was optimized into a FSC to be conversational and interactive chat to encourage probands to share information about their FH result with at-risk relatives. [file 12913_2023_9304_MOESM2_ESM.zip › Figure 2b_FSCR4.docx]

**Figure 3a. The Cascade Chatbot.**

**
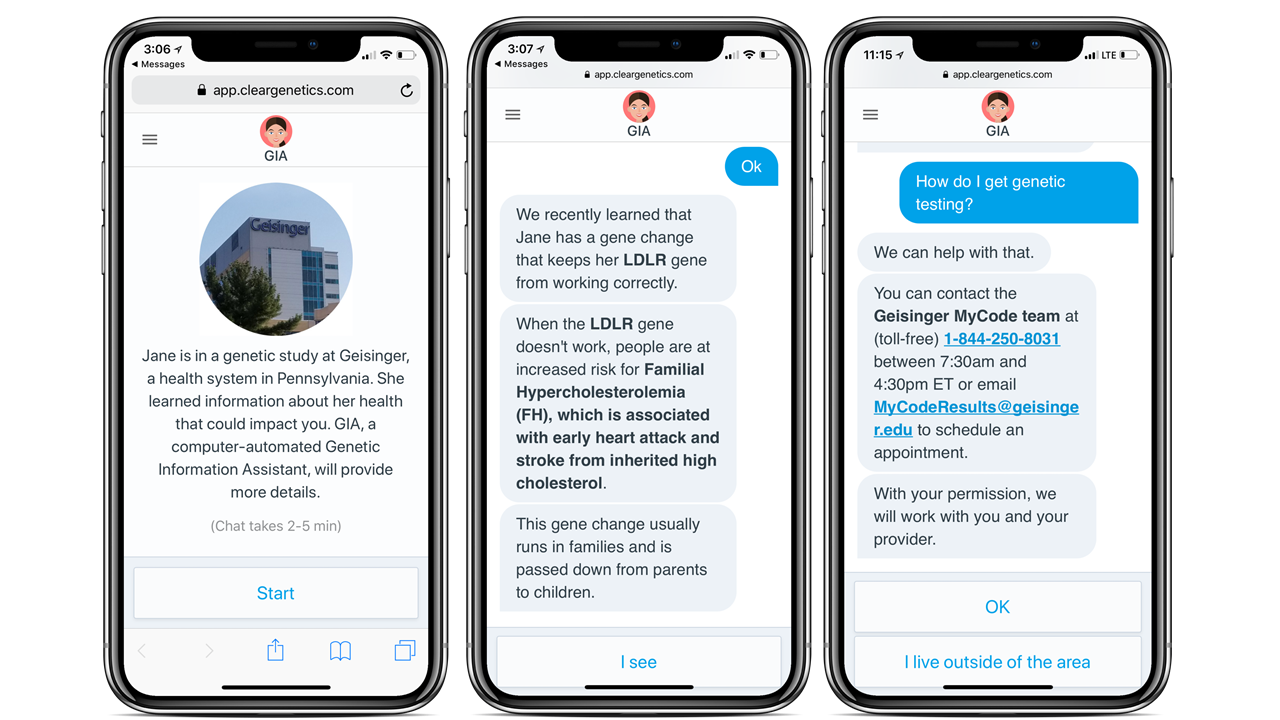
**

Supplement: Supplementary file 3 — Additional file 3: Supplemental Figure 3a. The Cascade Chatbot. The Cascade Chatbot is designed to share information about the proband’s FH result with at-risk relatives, provide the relative information about FH, and connect them with resources for cascade testing. Supplemental Figure 3b. The genetic testing ordering module additionto the Cascade Chatbot. The optimized Cascade Chatbot includes a module for at-risk relatives to order family variant testing through a mail-order genetic testing kit. [file 12913_2023_9304_MOESM3_ESM.zip › Figure 3a_Cascade ChatbotR4.docx]

**Figure 3b. The genetic testing ordering module addition to the Cascade Chatbot.**


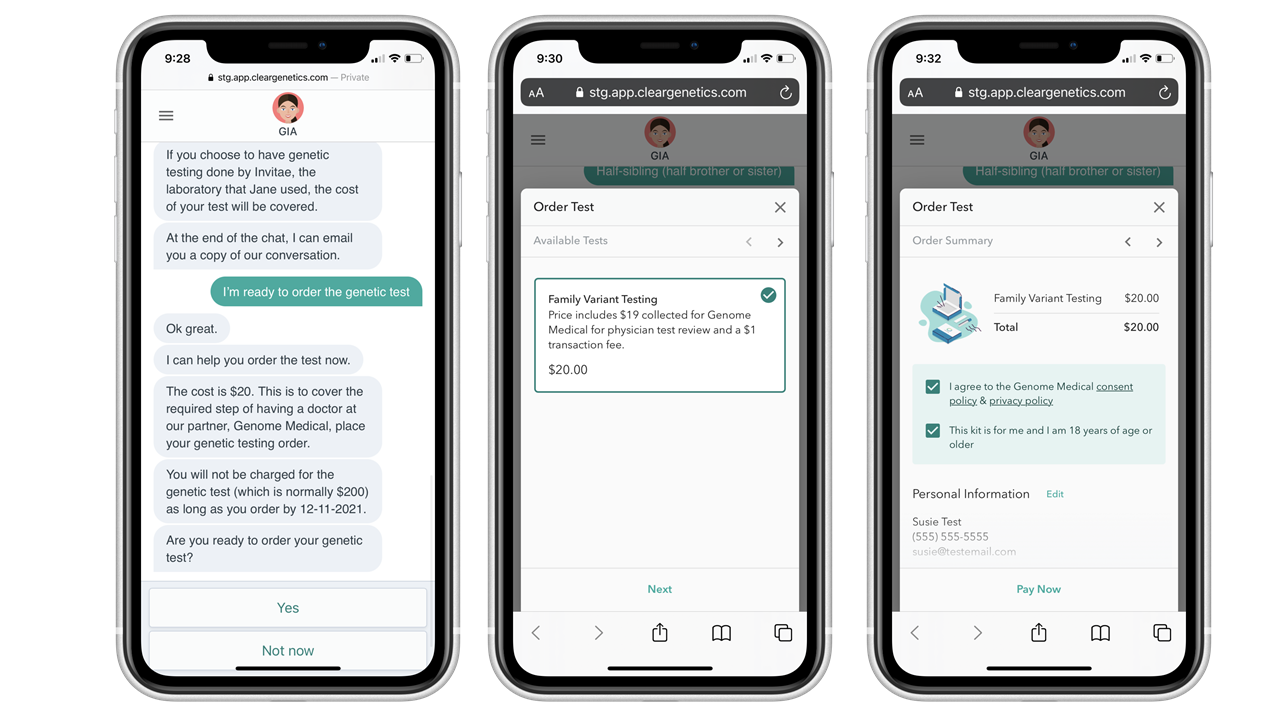

Supplement: Supplementary file 3 — Additional file 3: Supplemental Figure 3a. The Cascade Chatbot. The Cascade Chatbot is designed to share information about the proband’s FH result with at-risk relatives, provide the relative information about FH, and connect them with resources for cascade testing. Supplemental Figure 3b. The genetic testing ordering module additionto the Cascade Chatbot. The optimized Cascade Chatbot includes a module for at-risk relatives to order family variant testing through a mail-order genetic testing kit. [file 12913_2023_9304_MOESM3_ESM.zip › Figure 3b_Cascade chatbot ordering moduleR4.docx]
