# Supplementary material for: Optimizing communication strategies and designing a comprehensive program to facilitate cascade testing for familial hypercholesterolemia
Source: BMC Health Serv Res. 2023 Apr 5;23:340. doi: 10.1186/s12913-023-09304-y (PMC10074725; doi:10.1186/s12913-023-09304-y)
Supplement: Supplementary file 4 — Additional file 4: Supplemental Figure 4. Flyer for the FH Outreach and Support Program. The flyer is sent to probands in a packet they receive after receiving their FH result from MyCode and describes important points related to the direct contact program. [file 12913_2023_9304_MOESM4_ESM.pdf]

FH Outreach and Support Program

For Families with Familial Hypercholesterolemia (FH) and their Healthcare Professionals

How It Works

1. You found out that you have a genetic cause of FH.
2. You want help sharing this information with your family.
3. You give a genetic counselor contact information for your relatives and/or their healthcare professionals.
4. You give your relatives a “heads up” that they will be contacted.
5. The genetic counselor sends your relatives a “heads up” that they will be contacted.
6. The genetic counselor contacts your relatives and/or their healthcare professionals to discuss your FH result and next steps.

Why Would You Use This Program?

- Sharing complex health information about FH can be hard. A genetic counselor can help!
- Talking to a genetic counselor can motivate your at-risk relatives to get tested for FH.
- If relatives test positive for FH, they can get care for their FH health risks sooner.

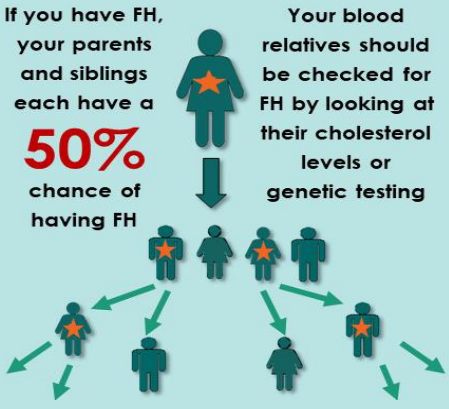

Image adapted from Centers for Disease Control and Prevention

Here’s What To Say When You Give Your Relative A “Heads Up”

“I learned that I have a serious genetic condition called FH, or inherited high cholesterol. FH runs in families. I want to protect your health and the health of our family members, so I asked a healthcare professional to contact you.”

“The healthcare professional is an expert in FH and can tell you more about it. They can explain the health risks for people like me who have FH, the treatments for FH, and how to get tested to learn if you have FH.”

“The healthcare professional can also tell you about testing options for FH and give you important information to share with your doctor. They will reach out soon by mail or email, first.”
