# Supplementary material for: Optimizing communication strategies and designing a comprehensive program to facilitate cascade testing for familial hypercholesterolemia
Source: BMC Health Serv Res. 2023 Apr 5;23:340. doi: 10.1186/s12913-023-09304-y (PMC10074725; doi:10.1186/s12913-023-09304-y)
Supplement: Supplementary file 5 — Additional file 5: Supplemental Figure 5. Primer Letter for the FH Outreach and Support Program. The Primer Letter template for relatives the proband chooses to be contacted directly by a genetic counselor as part of the FH Outreach and Support Program. [file 12913_2023_9304_MOESM5_ESM.pdf]

Geisinger mycode

{Today’s Date}

Dear {Relative’s Name},

I hope this letter finds you well. My name is {GC’s Name} and I am one of {Proband’s Name}’s healthcare professionals. {Proband’s Name} asked me to help share some important health information with you that may also affect your health and the health of your other family members.

**{Proband’s Name} was recently diagnosed with a serious genetic disorder called Familial Hypercholesterolemia (FH). FH is also known as inherited high cholesterol**. Having FH causes very high LDL (“bad”) cholesterol levels from birth. Having high levels of bad cholesterol puts one at a **higher risk for early heart disease, heart attack, stroke, and possibly even death, *if left untreated*.** Fortunately, there are many effective treatment options available.

As {Proband’s Name}’s {Relation to Proband}, **it is important to understand your own FH risks and options for next steps.**

**FH runs in families.** Based on your relation to {Proband’s Name}, you have a **__% chance** of also having inherited FH. Overall, parents, children, brothers and sisters of people with FH have a 50% chance of also having inherited FH or not. Other family members (aunts, uncles, nieces, nephews, cousins, grandchildren, etc.) may also have inherited this serious genetic disorder.

Since sharing complex health information can be hard, your relative gave me your contact information and asked that I reach out to you directly to discuss this information.

**I will call you soon to follow up on this information.**

**During our call, we will discuss more about:**

- {Proband’s Name}’s FH result.
- What this information means for you.
- Next steps you can take to protect your heart health and the health of your loved ones.

For more information about FH, you can visit **Geisinger’s FH webpage** at geisinger.org/FH and the webpage of the **FH Foundation**, a patient-centered organization for FH, at theFHfoundation.org.

If you do ***not*** wish to hear from me, please contact the Geisinger team (toll-free) at **1 (844) 250-8031** or **MyCodeResults@geisinger.edu** to let me know.

Please feel free to also contact me before I call with any questions or concerns you may have. Otherwise, I will talk to you soon!

Sincerely,

{GC’s Signature}
