# Supplementary material for: Optimizing communication strategies and designing a comprehensive program to facilitate cascade testing for familial hypercholesterolemia
Source: BMC Health Serv Res. 2023 Apr 5;23:340. doi: 10.1186/s12913-023-09304-y (PMC10074725; doi:10.1186/s12913-023-09304-y)
Supplement: Supplementary file 6 — Additional file 6: Supplemental Figure 6. IMPACT-FH Cascade Testing Program Workflow. Probands can choose multiple strategies for each of their at-risk relatives. All probands are provided with the Family and Healthcare Professional Packet and a flyer describing the FH Outreach and Support Program for direct contact after they receive their FH result even if they choose other communication strategies. [file 12913_2023_9304_MOESM6_ESM.docx]

**Figure 6. Snapshot of the workflow for the IMPACT-FH Cascade Testing Program**

**
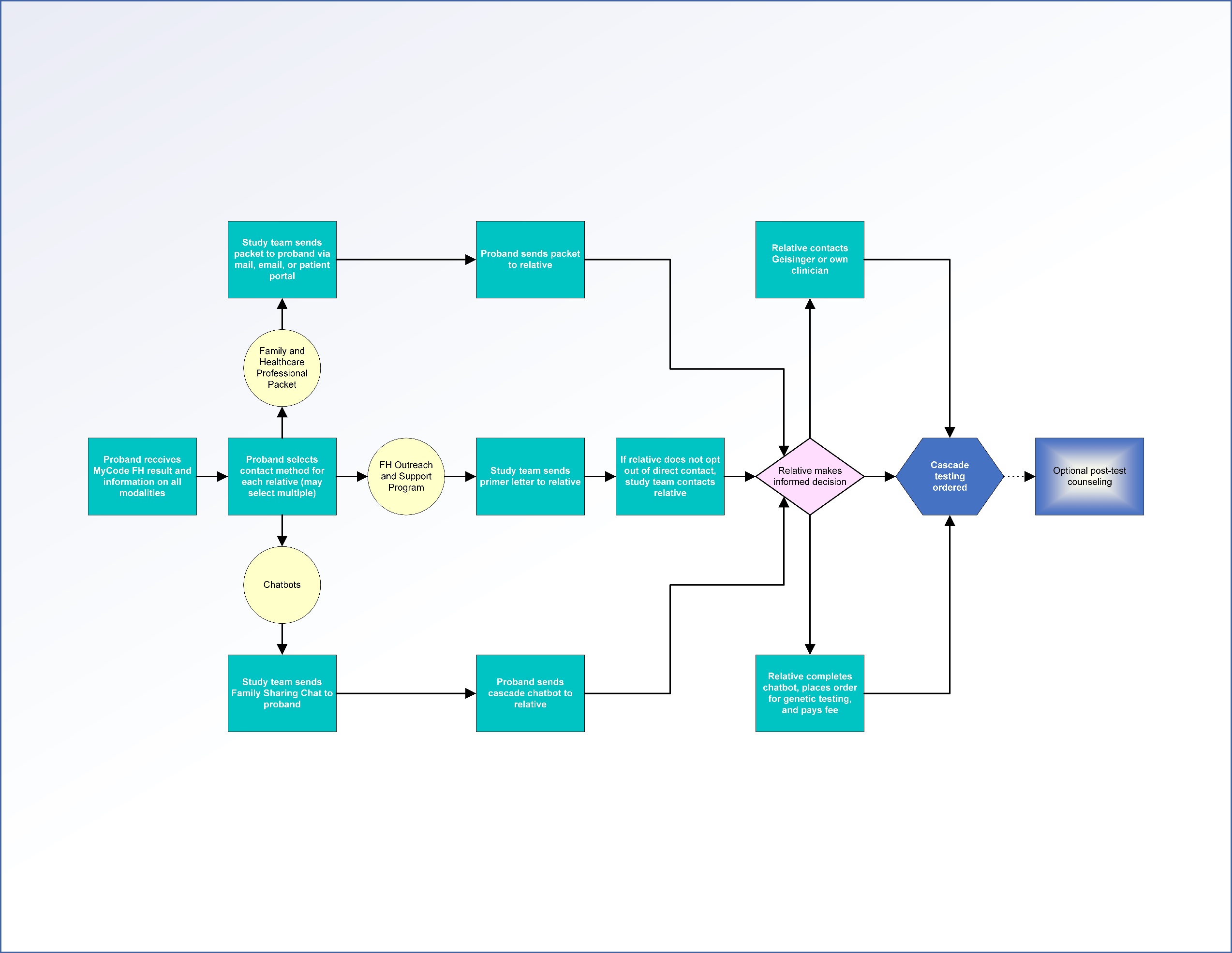
**

Note: Probands can choose multiple strategies for each of their at-risk relatives. All probands are provided with the Family and Healthcare Professional Packet and a flyer describing the FH Outreach and Support Program for direct contact after they receive their FH result even if they choose other communication strategies.
