# Supplementary material for: Optimizing communication strategies and designing a comprehensive program to facilitate cascade testing for familial hypercholesterolemia
Source: BMC Health Serv Res. 2023 Apr 5;23:340. doi: 10.1186/s12913-023-09304-y (PMC10074725; doi:10.1186/s12913-023-09304-y)
Supplement: Supplementary file 7 — Additional file 7. [file 12913_2023_9304_MOESM7_ESM.pdf]

IMPACT-FH: Family Member Survey

Welcome and Instructions

Greetings!

Geisinger, a health system in Pennsylvania, and the FH Foundation, a non-profit research and advocacy organization, want to learn how to better support people as they share information about Familial Hypercholesterolemia (FH) with their relatives.

Your responses will help us improve our current resources and design new ways for people to talk about FH with their family.

During the survey, you will be asked to:

· Review a letter
· Watch a video of a chatbot
· Read about a new program to help families share FH information
· Answer written response and multiple-choice questions about these resources

It should take you about 25 minutes to review the resources and complete the survey. We ask that you be as detailed as possible when answering the written response questions.

At the end of the survey, you will be invited to share this survey with your blood relatives and your spouse or partner.

Choosing not to take the survey will have no effect on your current medical care. If you choose to take the survey, you may exit it at any time.

Click the "Next" button at the bottom of the screen to start the survey.

If you have questions, concerns, or problems with the survey, please call our team at 1-866-910-6486, Option 2. You may also email us at IMPACTFH@geisinger.edu.

Thank you, we sincerely value your input.

IMPACT-FH: Family Member Survey

Demographic Information

Please answer the following questions.

* 1. How did you receive this survey?

- ◯ My family member, who is a Geisinger patient, sent me the survey
- ◯ My family member sent me the survey
- ◯ The FH Foundation sent me the survey
- ◯ The FH Foundation's social media page

* 2. Please type your age:

____

* 3. What is your biological sex?

- ◯ Male
- ◯ Female
- ◯ Prefer not to say

* 4. What is your annual household income?

- ◯ < $25,000
- ◯ $25 – 50,000
- ◯ $50 – 75,000
- ◯ $75 – 100,000
- ◯ > $100,000
- ◯ Prefer not to answer

* 5. What is your highest level of school completed?

- Some High School
- Graduated High School/GED
- Some College
- Associate’s Degree
- Bachelor’s Degree
- Graduate/Professional Degree
- Prefer not to answer

* 6. What state do you currently live in?

State/Province -- select state --

* 7. What is your relationship to the first person diagnosed with FH in your family? Please type:

If you were the first person diagnosed with FH in your family, please write "I was the first person."

* 8. Have you received testing for FH?

- Yes, doctor tested my cholesterol
- Yes, genetic testing
- No
- Unsure

IMPACT-FH: Family Member Survey

Demographic Information - FH Testing

Please answer the following questions.

* 9. Please type how old you were when you got tested for FH:

* 10. What was the result of the test?

- I have FH
- I do not have FH
- Unsure

IMPACT-FH: Family Member Survey

FH Infographic

Please review the infographic below to learn a little more about FH. This will help you understand questions on the survey. Click "Next" at the bottom of the page to proceed.

FH Infographic

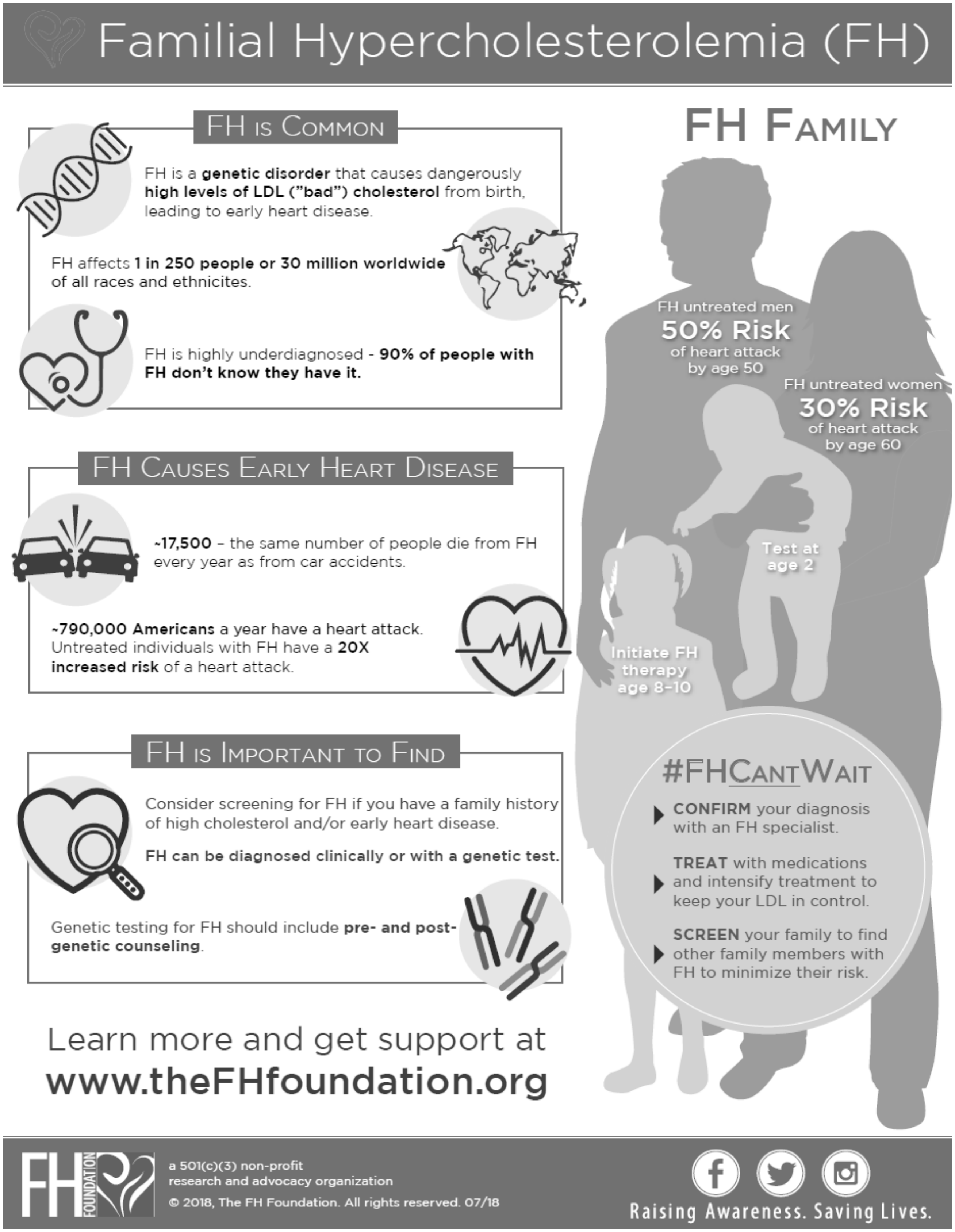

IMPACT-FH: Family Member Survey

Section 1: Dear Family Letter

The Dear Family Letter is a resource that was developed at Geisinger. It is given to people who receive a genetic diagnosis of FH through Geisinger's MyCode Community Health Initiative to help share their result with relatives of their choosing.

Please read the sample Dear Family Letter below before moving on to the next page.

You may return to this page at any time to review the Dear Family Letter by using the "Back" button at the bottom of the page. Your responses will be saved if you go back to review the letter.

mycode Geisinger

6/17/2020

Dear Ms. Jane Doe,

I learned that my *LDLR* gene does not work correctly through the Geisinger MyCode study. This gene change puts me at higher risk for early heart attack and stroke from inherited high cholesterol. Another name for this genetic risk is Familial Hypercholesterolemia (FH).

Gene changes run in families. Parents, children, brothers and sisters of people with a *LDLR* gene change have a 50% chance of having the same gene change and health risks. Other family members (aunts, uncles, nieces, nephews, cousins, grandchildren) may also have the same gene change and health risks.

People who have FH often need extra medical care. This extra care may include looking for and treating potentially life-threatening heart problems.

A simple “yes/no” blood or saliva test can tell you if you also have the same gene change and health risks.

You may be able to get this “yes/no” test for free or at lower-cost from the same lab that ran my test. Free genetic testing for family members is offered for 90 days after **6/10/2020**, my report date. The appointment with the provider to order this test will be billed to you or your insurance.

What you should do next:

- **Call the Geisinger MyCode Genomic Screening and Counseling team (toll-free) at 1-844-250-8031.** They can schedule an appointment to start the “yes/no” testing process. They can also answer your questions.

- **If you do not live in PA, you can find a genetic counselor to order this test and discuss your risks at:** https://www.nsgc.org/page/find-a-genetic-counselor

The healthcare provider ordering your test will need this information:

c.2054C>T p.Pro685Leu in the *LDLR* gene (NM_000527.5)
Laboratory: Invitae.

This letter gives my permission for Geisinger to share my genetic test results with you for your care. **Please bring this letter with you to your appointment.**

If you have any questions or concerns, please call the Geisinger MyCode team (toll-free) at **1-844-250-8031**.

Sincerely,

____

Mr. John Doe

INVITAE

SECONDARY FINDINGS SCREEN RESULTS

**Patient name:**	John Doe	**Sample type:**	gDNA	**Report date:**	
**DOB:**		**Sample collection date:**		**Invitae #:**	
**Sex:**	Male	**Sample accession date:**		**Clinical team:**	
**MRN:**					

Test performed

Sequence analysis and deletion/duplication testing of the 59 genes listed in the Genes Analyzed section.

- Secondary Findings Add-on

RESULT: POSITIVE

A clinically significant genetic change was found in the LDLR gene, which is associated with a heart-related condition.

GENE	VARIANT	ZYGOSITY	VARIANT CLASSIFICATION
LDLR	c.2054C>T (p.Pro685Leu)	heterozygous	PATHOGENIC

About this test

This test evaluates 59 genes for variants (genetic changes) that indicate a significantly increased risk of developing certain types of cancer, heart-related conditions, or other types of actionable medical genetic conditions. These are disorders for which effective medical interventions and preventive measures are known and available. Genetic changes of uncertain significance are not included in this report; however, if additional evidence becomes available to indicate that a previously uncertain genetic change is clinically significant, Invitae will update this report and provide notification.

Next steps

- This is a medically important result that should be discussed with an appropriate healthcare provider. Genetic counseling is recommended to discuss the implications of this result and potential next steps.
- Consider sharing this result with relatives as they may also be at risk. Details on our Family Variant Testing program can be found at www.invitae.com/family.
- Register your test at www.invitae.com/patients to download a digital copy of your results. You can also access educational resources about how your results can help inform your health.

Clinical Summary

A Pathogenic variant, c.2054C>T (p.Pro685Leu), was identified in LDLR.

Laboratory Director Tina Hambuch, Ph.D., FACMG
NY Laboratory Director Swaroop Aradhya, Ph.D., FACMG
Invitae 1400 16th Street, San Francisco, CA 94103
E: clientservices@invitae.com P: 415.374.7782 or 800.436.3037

FH FOUNDATION | Geisinger

IMPACT-FH: Family Member Survey

Section 1: Dear Family Letter

Please be detailed in your responses to the below questions, so we can better understand how to improve our resources for individuals and families with FH.

You may use the "Back" button at the bottom of the page to return to the sample Dear Family Letter. Your responses will be saved if you go back to review the letter.

* 14. What would you do if you received this letter from your relatives?

* 15. What other information would you want from a letter like this?

* 16. What can we do to improve this letter to help you or your relatives who may have FH take next steps to find out if you or they have FH?

17. What else do you want to share with us about the letter and/or how to make it better?

IMPACT-FH: Family Member Survey

Section 2: Chatbot

A chatbot is an online conversational tool. People with FH are offered a chatbot to help them more easily share information about FH with relatives. Relatives of the individual's choosing are sent the chatbot to help them learn more about FH and their risks.

Please watch the chatbot video below before moving on to the next page.

If you are having trouble playing this video within the survey, please copy and paste the following link *in a new tab or window*: https://youtu.be/aTDKdodrIzs

You may return to this page at any time to re-watch the chatbot video by using the "Back" button at the bottom of the page. Your responses will be saved if you go back to re-watch the video.

IMPACT-FH: Family Member Survey

Section 2: Chatbot

Please answer the following question.

You may use the "Back" button at the bottom of the page to return to the chatbot video. Your responses will be saved if you go back to re-watch the video.

* 19. Why would you use the chatbot if a relative sent it to you? *Please be detailed in your response.*

IMPACT-FH: Family Member Survey

Section 2: Chatbot

Please answer the following question.

You may use the "Back" button at the bottom of the page to return to the chatbot video. Your responses will be saved if you go back to re-watch the video.

* 20. Why would you NOT use the chatbot if a relative sent it to you? *Please be detailed in your response.*

IMPACT-FH: Family Member Survey

Section 2: Chatbot

Please answer the following question.

You may use the "Back" button at the bottom of the page to return to the chatbot video. Your responses will be saved if you go back to re-watch the video.

* 21. Why are you unsure about whether you would use the chatbot if a relative sent it to you? *Please be detailed in your response.*

IMPACT-FH: Family Member Survey

Section 2: Chatbot

Please be detailed in your responses to the below questions, so we can better understand how to improve our resources for individuals and families with FH.

You may use the "Back" button at the bottom of the page to return to the chatbot video. Your responses will be saved if you go back to re-watch the video.

* 25. What can we improve about the chatbot to help you or your relatives who may have FH take next steps to find out if you or they have FH?

26. What else do you want to share with us about the chatbot and/or how to make it better?

IMPACT-FH: Family Member Survey

Section 3: Direct Contact Program

The Direct Contact Program is a new program being designed by Geisinger and the FH Foundation. It will be another way for individuals diagnosed with FH to share their diagnosis with the relatives of their choosing. The Direct Contact Program has not been finalized yet. The description below is a general idea of what the program will look like.

Please read the information below before moving on to the next page.

You may return to this page at any time to review this information by using the "Back" button at the bottom of the page. Your responses will be saved if you go back to review this information.

What is a Direct Contact Program?

Step 1

A person gets tested, via genetic testing and/or cholesterol testing, and finds out they have Familial Hypercholesterolemia (FH).

Step 2

The person with FH gives a healthcare provider permission to share their FH diagnosis with their at-risk relatives.

- The person with FH can pick which at-risk relatives they want the healthcare provider to contact and gives the healthcare provider contact information (for example, address, email, telephone number) for each of those relatives.

Step 3

The healthcare provider contacts at-risk relatives to share that their family member has FH and to explain their risks of heart disease if they have FH, too.

Step 4

A healthcare provider, like a genetic counselor or doctor, can counsel relatives and help them get testing.

Why would someone use a Direct Contact Program?

Sharing complex health information about FH with at-risk relatives can be hard.

Talking to a healthcare provider can motivate at-risk relatives to get tested for FH.

If relatives test positive for FH, they can get care for their FH health risk sooner.

Section 3: Direct Contact Program (CASCADE)

CASCADE is a new program we are starting with the Direct Contact Program. CASCADE offers genetic counseling, information and support to help individuals consider getting tested for FH. The program also allows family members to order a genetic test kit to their home address, to complete and send back. Family members could choose this option in talking to a provider doing direct contact and/or in a chatbot.

Please read the information below about the CASCADE program and answer the following questions.

"Cascade Testing" for At-Risk Relatives

Geisinger is designing a program, called CASCADE, to help at-risk relatives get cascade testing.

- CASCADE: Contact And Support, Counseling, And DNA Testing Empowerment

What is cascade testing?

- When at-risk relatives get tested for FH, through genetic testing or cholesterol testing, after the first person in a family is diagnosed.

What does CASCADE do?

- Provides genetic counseling for at-risk relatives
- Helps at-risk relatives get genetic testing for FH using a mail-order kit
- Helps at-risk relatives connect with a doctor for cholesterol testing

How much does CASCADE cost?

- After a person receives a positive FH genetic test result, there is a period of time when at-risk relatives can have no-cost genetic testing
- A small fee of $20 will be required for a doctor to order the genetic test
- Testing company works with insurance for testing outside of the no-cost window – self-pay option is $200

IMPACT-FH: Family Member Survey

Section 3: Direct Contact Program

Please answer the following questions.

You may use the "Back" button at the bottom of the page to return to the information about the Direct Contact Program or CASCADE. Your responses will be saved if you go back to review the information.

* 33. Ideally, if a healthcare provider were to contact you or your realtives who may have FH to share information about an FH diagnosis and the risks, what would you want them to say/do?

* 34. How would your other relatives respond if a healthcare provider contacted them to share information about a common relative's FH diagnosis and their risks?

35. What else do you want to share with us about the Direct Contact Program and/or how to make it better?

IMPACT-FH: Family Member Survey

Additional Opportunities

* 36. Would you be interested in taking part in further opportunities on how healthcare providers can support families talking about FH?

- Yes
- No

IMPACT-FH: Family Member Survey

Additional Opportunities - Contact Information

* 37. Please provide your name, phone number and email address so we can contact you with more information on ways to take part in more FH projects:

Name

Email Address

Phone Number

Share a Survey with your Family Members!

We are also interested in understanding how *your family members* feel about these materials.

If you are willing to share this survey with your blood relatives, or your spouse/partner, *please copy the link below and share it with them*.

You may share the link with your family members in any way you choose.

Family Member Survey Link: [Link to Family Member Survey]
