# Supplementary material for: Optimizing communication strategies and designing a comprehensive program to facilitate cascade testing for familial hypercholesterolemia
Source: BMC Health Serv Res. 2023 Apr 5;23:340. doi: 10.1186/s12913-023-09304-y (PMC10074725; doi:10.1186/s12913-023-09304-y)
Supplement: Supplementary file 10 — Additional file 10. [file 12913_2023_9304_MOESM10_ESM.docx]

Alignment with the 21 Standards for reporting qualitative research (SRQR)

| Item | Alignment with Manuscript | Explanation |
| --- | --- | --- |
| 1. Title | 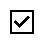 | Provides a concise description of the nature of the topic, but title does not indicate the study is qualitative or data collection methods (p.1) |
| 1. Abstract | 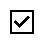 | Summarized of key elements including background, purpose, methods, results, and conclusions (p.3) |
| 1. Problem Formulation | 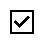 | Described the problem/phenomenon being studied and its significance using relevant theory and empirical work (p.4-5) |
| 1. Purpose or research question | 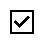 | End of introduction provides a paragraph on the purpose of the study and its objectives (p.6) |
| 1. Qualitative Approach and Research Paradigm | 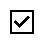 | The qualitative approach was described, and guiding theory was cited (p.6) |
| 1. Researcher characteristics and reflexivity | 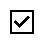 | The transdisciplinary team collecting and analyzing the data were described to identify their qualifications, experience, and connections to the study (p.9) |
| 1. Context | 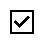 | Setting and salient contextual factors were described (p.6-8) |
| 1. Sampling strategy | 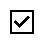 | Authors described how and why research participants were selected and how sample saturation was determined (p.7-9) |
| 1. Ethical issues pertaining to human subjects | 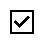 | Explanation of approval by appropriate ethics review board and consent are described in the declarations at the end of the manuscript (p.22) |
| 1. Data collection methods | 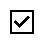 | Types of data collected were described as well as data collection procedures, dates of collection, triangulation of methods, and the iterative analysis process (p.6-9) |
| 1. Data collection instruments and technologies | 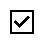 | Interview guides and surveys were described as well as their procedures. These procedures did not change over the course of the study (p.6-9) |
| 1. Units of study | 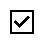 | We report the number of participants per method and provide participant descriptions to characterize the sample (p.9) |
| 1. Data processing | 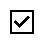 | We described data processing methods related to transcription, data management and verification, and coding (p.6-9) |
| 1. Data analysis | 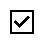 | We described the processes for coding and thematic analysis. We described the levels of coding, what team members were involved, and guiding paradigms/approaches (p.8-9) |
| 1. Techniques to enhance trustworthiness | 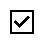 | The combination of methods allowed for triangulation, which is described (p.5). Authors and the coding team also met regularly to ensure trustworthiness of analysis (p.8-9) |
| 1. Synthesis and interpretation | 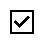 | Main findings are reported in text and in further detail in tables (p.9-13) |
| 1. Links to empirical data | 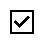 | In tables containing results, we provide the evidence (quotes) to support the themes and describe the team’s Traffic Light approach and why suggested optimizations were categories as green, yellow, or red |
| 1. Integration with prior work, implications, transferability, and contributions(s) to the field | 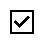 | The Discussion provides a short summary of main findings, how findings connect to support earlier scholarship and develop new communication strategies. We discuss the transferability of findings and the unique contributions to scholarship (p.13-18) |
| 1. Limitations | 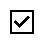 | In the Discussion, we provide a detailed breakdown of this study’s limitations (p.17) |
| 1. Conflicts of interest | 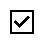 | At the end of the manuscript, we provide potential sources of influence (p.22) |
| 1. Funding | 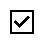 | We provide the source of funding for this research and the role of the funders (p.22) |
