# Supplementary material for: Optimizing communication strategies and designing a comprehensive program to facilitate cascade testing for familial hypercholesterolemia
Source: BMC Health Serv Res. 2023 Apr 5;23:340. doi: 10.1186/s12913-023-09304-y (PMC10074725; doi:10.1186/s12913-023-09304-y)
Supplement: Supplementary file 11 — Additional file 11. [file 12913_2023_9304_MOESM11_ESM.docx]

**IMPACT-FH - Aim 2, Phase 1: Quality Improvement Dyadic Interview Guide**

**Introduction**: Thank you for agreeing to help with this quality improvement process. As a reminder, your participation in helping us improve the quality of our care and resources is voluntary. As you recall, we expect this interview to take 45 minutes to an hour. Your responses will help us improve how we communicate with individuals and families about risks related to FH. I will ask you some broad questions and then follow up with some more specific questions about the materials you were sent for homework (the “Dear Family” letter, chatbots, and description of the Direct Contact Program). Have you each reviewed the materials that were sent before to your emails?

If yes: proceed to next section.

If no: need to reschedule until they have reviewed the materials.

Everything you tell me will be kept confidential. This means that your interview responses will only be shared with research team members. When we write our report on this and the other interviews we are conducting, nothing in our report will identify you. Please be honest with your responses. You can say whatever you want – nothing will hurt my feelings and nothing you say will have a negative effect on your care. We will record this conversation, but the transcript from the conversation will not include any information that identifies you. Please remember: you don’t have to talk about anything you don’t want to talk about. You can decline to answer any question and you may end the interview at any time.

Is it OK if I start recording now?

| **Stem** | **Probe** | **Concept/Modality** |
| --- | --- | --- |
| - How did your family talk about your FH diagnosis? | - (to proband) How did you share your FH results with (family member name) in the interview today? - (to family member) How did you respond when (proband name) told you about this? | Warm up – Disclosure of Result |
| - How does your family talk about their FH diagnosis? (e.g., risks for heart disease, having or managing high cholesterol, passing the gene down, etc.) | - How does your family feel about your/their FH diagnosis? - What’s made it easier to share this information with family members? - What’s made it difficult to share this information with family members? | Family Health Communication of FH Norms |
| - If you were looking for resources or information on FH to help you talk to family members about FH, where would you look for these resources? | - What information would you want to share with your relatives who may have inherited FH? | Information Needs |
| *[Description of Direct Contact - Stress that proband has to give consent, relative’s name and contact info, and provider would only contact those the proband wanted]*   - What do you think about having a provider contact relatives directly to talk about your FH result? | - What type of provider would you want to do this? (e.g., nurse, genetic counselor, PCP/GP, navigator trained for this purpose etc.)   - Can you explain what makes you choose that provider?   - How would you feel about a trained representative or healthcare provider from an organization like the FH Foundation contacting relatives about your FH result? (may need to explain FH Foundation) - Think about how you and your family members communicate about FH. What would be the best thing for your [preferred provider] to say when they contact your family members to discuss FH?   - Is there information a provider should NOT share that should only come from a relative?   - How would you (FH proband) want to be involved with a provider sharing this information with family members? (e.g., go over script of what to tell relative with provider, discuss when to contact family members, etc.) - What should a provider say to at-risk family members to prompt action? - What should a provider NOT say to at-risk family member to prompt action? | Direct Contact |
| - How would family members respond if they were contacted by a provider? | - Which of your family members would respond best to this method of contact? (e.g., specific people in family and/or certain family relationships like siblings, children, etc.) - Why might this work best for those family members? - Who would this NOT work for in your family?   - Can you tell me more about why this might not work [person/people]? | Direct Contact |
| - Ideally, if a provider were to help share this information with family – what should the process be? what would you want this to look like? | - How would you feel if a provider sent a letter first and then followed up with a phone call for the Direct Contact Program? (letter would be different from “Dear Family” letter) - What approach might work better for you/your family? - What contact information for relatives would you be willing to provide? (e.g., phone number, email address, mailing address, etc.) - What would make it easier for family members to do follow up testing? - How else would you want a provider to help? | Direct Contact |
| *[Describe CASCADE program]*   - Geisinger has a program called CASCADE that you read a little material on. This program include genetic counseling and allows family members to order an FH genetic test via a mail order kit to do at home and send back. Family members could choose this option in talking to a provider during Direct Contact. If this was offered to you (family members) how would you respond? | - How would other family members respond to this option? - How can we improve how we talk about this program with at-risk family members?   - How would you feel if you had the option to order a genetic test (mail order kit)?   - How would you want to order a genetic test (mail order kit)? (e.g., have provider order for you, order online, etc.) - What other information on this option would you want as a proband/relative? | Direct Contact |
| *Transition to Letter* | Thank you for sharing your thoughts on a Direct Contact program. We’d now like to switch topics slightly and get your thoughts on the “Dear Family” Letter. This was sent to you via email. It may help if you are able to look at it or your notes on it while we go through questions. |  |
| - (To proband) How would you feel using this letter to help you share your results? | - Please tell me more about why/why not. - How would you use it? (e.g., mail it, use it to guide conversation, etc.) | “Dear Family” Letter |
| - (To family member) How would you feel receiving this letter? | - What would you do next if you got this letter? (e.g., talk to doctor, talk to family, etc.) - What can we change about the letter to help you talk about your relative’s result with your doctor?   - What could we improve about the letter to make you and other family members follow up with testing? - What questions would you ask your relative if you received this letter? | “Dear Family” Letter |
| - (To Both) How could we make the letter better? | - What can we improve to make it more likely someone will take action to follow up and pursue testing? - What other information would you want from the letter? - Can you explain what type of information that is? Why? | “Dear Family” Letter |
| *Transition to Chatbot* | Thank you for sharing your thoughts on the “Dear Family” Letter. We’d now like to move to our last topics and get your thoughts on the chatbots. The links for the chatbots were sent to you via email. It may help if you are able to look them or your notes on them while we go through questions. |  |
| - (To proband) How would you feel about using a chatbot to share your results? | - Please tell me more about why/why not. - If you were to use a Chatbot to share your results with family, how would you do that? (i.e., how to send, timing of sending, contacting relative before sending, etc.) | Chatbot |
| - (To family member) How would you feel receiving the chatbot? | - What questions would you ask of your relative if you received this? - What would you do next if you got the chatbot? (e.g., talk to doctor, talk to family, look up info online, etc.) - How comfortable would you be if after the chatbot gave information on FH, it gave you the option to order a genetic test by mail? - How can we improve/enhance the chatbot to help you talk about your relative’s result with your doctor? | Chatbot |
| - (To both) How could we make this tool better for both of you? | - How would you feel if you received reminders from the chatbot on this information?   - How often would you want reminders?   - How many reminders would be too many?   - When should these reminders come in? - What other information would you want from the chatbot?   - Is there information that should NOT come from the chatbot and only from a healthcare provider? Relative?     - Can you explain what type of information that is? Why? | Chatbot |
| - How would you feel having a combination of resources, like the letter, chatbot, and provider help to share this information? | - What combination do you think is best? - Would you want to use different communication resources at different times as you share this information with family? - Would you use different communication resources with different family members? If so, How? - As a family member, how would you respond to receiving a combination of these resources?   - As a family member, what would work best for you? - Please tell me more about why X combination would work best. | Combination of Modalities |
